# Identification and verification of diagnostic biomarkers related to endoplasmic reticulum stress for atherosclerosis

**DOI:** 10.1186/s12872-026-05745-5

**Published:** 2026-03-20

**Authors:** Xiaoyun Liu, Huimin Pang, Xuediao Pan, Shuxian Fan, Yi Deng, Mengdie Yu, Xinyuan Xie, Yufang Pan

**Affiliations:** 1 School of Pharmacy, Guangdong Pharmaceutical University, Guangzhou, 510006 China; 2 The Third People’s Hospital of Longgang District, Shenzhen, 518115 China

**Keywords:** Atherosclerosis, Endoplasmic reticulum stress, Biomarker, Single cell analysis

## Abstract

**Background:**

Endoplasmic reticulum stress (ERS) is linked to the progression of atherosclerosis (AS). The function of ERS-related genes (ERSRGs) in AS remains ambiguous, hence, this study sought to examined their association with AS.

**Methods:**

Datasets relevant to AS were acquired from the Gene Expression Omnibus (GEO) database. Initially, differentially expressed genes (DEGs) were identified between AS and controls. Weighted gene co-expression network analysis (WGCNA) derived AS-related module genes. The intersection of DEGs, AS-related module genes, and ERSRGs was subsequently evaluated by three machine learning algorithms. After processing expression analysis and receiver operating characteristic (ROC) curve evaluation, biomarkers were ultimately found. A nomogram was developed and validated to predict the incidence of AS based on biomarkers. Finally, single cell RNA sequencing (scRNA-seq) analysis was utilized to identify distinct cell subpopulations, followed by cell communication and pseudotime analysis.

**Results:**

ANKRD1, BDNF, HLA-B, NLRP3, NOD2, and XAF1 were recognized as biomarkers for AS. The nomogram developed using biomarkers demonstrated exceptional efficacy in predicting AS occurrence. The scRNA-seq analysis annotated 6 cell subpopulations, including NK cells, B cells, T cells, vascular smooth muscle cells (VSMCs), endothelial, and macrophages. VSMCs exhibited increased communication in AS samples compared to control samples. Moreover, we noted that HLA-B exhibited elevated expression during the prophase and anaphase of VSMCs differentiation, whereas XAF1 had increased expression in the later stages of VSMCs differentiation.

**Conclusion:**

This study found 6 biomarkers (ANKRD1, BDNF, HLA-B, NLRP3, NOD2, and XAF1) associated with ERS in AS, providing novel diagnostic targets for the condition.

**Supplementary Information:**

The online version contains supplementary material available at 10.1186/s12872-026-05745-5.

## Introduction

Atherosclerosis (AS), the predominant cardiovascular disease (CVD), is chiefly induced by lipid deposition and inflammation in major arteries, which eventually may lead to the occurrence of its clinical complications, myocardial infarction (MI) and stroke [1]. In the last 30 years, the molecular pathways underlying AS development have been thoroughly investigated. Therapeutic advances, including statins, PCSK9 inhibitors, and anti-inflammatory agents also have been demonstrated as the most efficacious method for the prevention and treatment of AS [2]. Despite these progress, it continues to be the predominant cause of mortality globally [3]. This underscores limitations in current biomarkers to predict individual risk or guide precision therapies, emphasizing the urgent need for novel biomarkers to decode AS heterogeneity and enable early intervention.

Endoplasmic reticulum (ER) is a crucial organelle that facilitates the correct folding of polypeptides and proteins [4]. Disruption of the ER’s protein-folding capability results in the buildup of unfolded and misfolded proteins, culminating in endoplasmic reticulum stress (ERS) [5]. In response to ERS, cells activate multiple adaptive mechanisms, such as the unfolded protein response (UPR), ER-associated degradation (ERAD), and reticulophagy, are initiated to reestablish protein homeostasis. Nonetheless, sustained activation can result in a maladaptive cellular response, contributing to various diseases such as cancer, kidney diseases, fatty liver disease, diabetes, obesity and cardiovascular diseases (CVDs) [6]. Emerging evidence suggests that ERS influences tumorigenesis, metastasis, and resistance to therapy [7]. Previous studies have also identified elevated ERS markers were found in human coronary artery lesions, such as BiP and CHOP, indicating a link between ERS and atherogenesis [5]. Despite this, clinical validation of ERS-targeted therapies remains limited, primarily due to a lack of preclinical models that accurately reflect the complexity of human diseases, and the precise molecular mechanisms in ERS-mediated AS remain incompletely defined.

In this study, we used bioinformatics analysis to identify biomarkers associated with ERS in AS and developed a nomogram to assess their potential in predicting the onset of AS. Importantly, ERS contributes to atherosclerosis mainly by promoting inflammation, immune activation, and stress-induced apoptosis [8]. Therefore, genes involved in these biological processes may act as critical molecular links between ERS and AS. Additionally, we explored the mechanisms by which these biomarkers influenced AS through immune infiltration analysis, functional enrichment, and regulatory networks evaluation. Furthermore, we also investigated the conditions of AS at single-cell level. The discovery of novel biomarkers not only facilitates the determination of genes associated with the pathogenesis and progression of AS, but also offers promising opportunities for early intervention and therapeutic strategies.

**Fig. 1:**
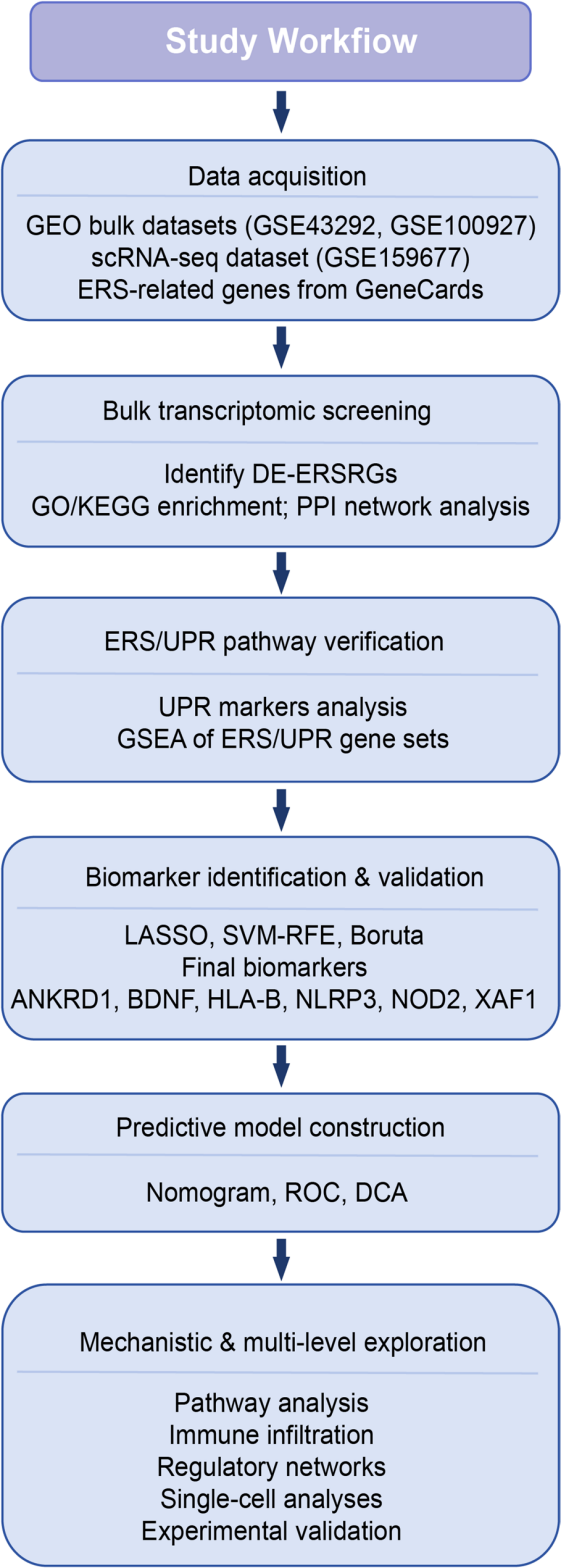
Research workflow

The flowchart of this study is shown in Fig. 1.

## Materials and methods

### Data acquisition

Transcriptome datasets related to AS (GSE43292, GSE100927, and GSE111782) and single-cell RNA sequencing (scRNA-seq) dataset GSE159677 were obtained from GEO database (http://www.ncbi.nlm.nih.gov/geo/). The GSE43292 dataset (GPL6244) comprised 64 carotid artery tissue samples derived from 32 patients undergoing carotid endarterectomy, with paired samples collected from each individual, including an AS and a distant macroscopically intact carotid tissue (control) (32 paired samples in total). This within-subject paired design partially controls for stable individual-level characteristics and shared medication exposure. This dataset was used as the training cohort. The validation dataset GSE100927 (GPL17077) included 29 atherosclerotic carotid lesion samples and 12 control carotid artery samples. An additional validation set, GSE11782 (GPL571), included carotid plaque samples from 9 symptomatic patients and 9 asymptomatic patients. All comparisons between cohorts were performed at the model-performance level rather than through joint expression-level analyses. The GSE159677 dataset comprised 3 pairs of AS and control tissue samples. Furthermore, 1,210 endoplasmic reticulum stress-related genes (ERSRGs) were obtained from GeneCards database (https://www.genecards.org/) with relevance score ≥ 1.

### Differential expression analysis

The “limma” package (version 3.54.2) [9] was used to screen differentially expressed genes (DEGs) between AS and control samples in training set. The screening threshold was *P* < 0.05 and |log_2_fold change (FC)| > 0.5, and results were shown by volcano plot which was drafted by “ggplot2” package (version 3.54.2). Top 20 up- and down-regulated DEGs of |log_2_FC| were displayed by heat map, drafting by “pheatmap” package (version 1.0.12). All normalization and differential expression analyses were conducted within each dataset independently, without direct pooling or cross-platform batch correction.

### Weighted Gene Co-expression Network Analysis (WGCNA)

WGCNA was performed to screen genes that closely correlated with AS. In training set, samples were clustered to examine whether outlier samples were existed, and then eliminated. To ensure gene interactions followed a scale-free distribution, the optimal soft threshold (β) was selected at R^2^ close to 0.85 and mean connectivity near 0. Dynamic tree cutting approach was utilized to separate modules, each containing a minimum of 200 genes. AS and control samples were utilized as traits, pearson analysis was processed to evaluate the correlation of obtained modules with traits. Ultimately, gene modules exhibiting the highest correlation coefficient with traits were designated as important modules (*P* < 0.05). Genes in important modules were defined as AS-related module genes.

### Screening and analyses of Differentially Expressed-ERSEGs (DE-ERSEGs)

DEGs, AS-related module genes, and ERSRGs were overlapped, and then visualized by “ggvenn” package (version 0.1.10) [10]. The intersection of these genes was designated as DE-ERSRGs. To make further efforts for informing molecular functions and effect mechanism of DE-ERSRGs, Gene Ontology (GO) and KEGG: Kyoto Encyclopedia of Genes and Genomes (KEGG) enrichment analyses were performed by “clusterProfiler” package (version 4.6.2) [11] with adj.*P* < 0.05. Top 5 of each part of GO terms and top 10 KEGG pathways were visualized by “ggplot2” package (version 3.5.1) [12]. Afterthat, the interaction of DE-ERSRGs in protein level was explored by STRING database (https//string-db.org/), and was presented with a Protein-protein interaction (PPI) network.

### Identification of biomarkers in AS

After obtaining DE-ERSRGs, machine learning algorithms including least absolute shrinkage and selection operator (LASSO), support vector machine-recursive feature elimination (SVM-RFE), and Boruta were carried out to screen feature genes utilizing “glmnet” (version 4.1-8) [13], “e1071” (version 1.7–14), and “Boruta” package (version 8.0.0), respectively.

For LASSO regression, a binomial deviance loss function was used with α = 1, and the regularization parameter λ was determined by 5-fold cross-validation repeated 10 times using the 1-standard-error rule to select features with non-zero coefficients. SVM-RFE was implemented with a linear-kernel support vector machine, and the cost parameter C was selected from {0.1, 1, 10} via grid search nested within 5-fold cross-validation. Boruta feature selection, based on a random forest framework, was performed with 300 iterations to identify all relevant features.

Feature genes identified by the three algorithms were intersected to define candidate biomarkers. Subsequently, the expression levels of candidate biomarkers were evaluated, and their diagnostic performance was assessed using receiver operating characteristic (ROC) curve analysis. Genes showing significant differential expression between AS and control samples (*P* < 0.05) and an area under the ROC curve (AUC) greater than 0.6 were retained and defined as final biomarkers for AS.

### Construction of nomogram

Based on biomarkers, a nomogram was created using “rms” package (version 6.7-0) to forecast AS’s occurrence. Each biomarker was associated with a score, the aggregate of these scores constituted total points. The diagnosis likelihood of the disease was projected based on total points. A greater total of points correlated with an increased chance of sickness. Then, nomogram’s predictive function was evaluated by calibration curve, and the validity was analyzed by ROC curve.

### Gene Set Enrichment Analysis (GSEA) and Gene Set Variation Analysis (GSVA)

To find corresponding functions of biomarkers involved in the development of AS, GSEA was performed. First, spearman analysis was conducted on biomarkers with all genes by “psych” package (version 2.3.3) [14]. Then, genes were prioritized according to their correlation coefficient. Third, sing gene GSEA was performed using “clusterProfiler” package with KEGG background gene set c2.cp.kegg_medicus.v2023.2.Hs.symbols.gmt downloading from GSEA website (http://www.gsea-MSigdb.org/gsea/msigdb) (|NES| > 1, adj.*P* < 0.05). Subsequently, to understand the pathways that were activated or inhibited in AS samples, GSVA was conducted with KEGG background gene set. GSVA score of each pathway was calculated via the “GSVA” package (version 1.46.0) [15], and the disparities between AS and control groups were examined utilizing “limma” package (|t| > 2, *P* < 0.05). Top 10 activated or inhibited pathways of t value were displayed by heat map. For pathway-level analyses, enrichment results were interpreted with consideration of multiple testing and overall pathway consistency, rather than relying solely on nominal *P* values.

### Regulatory network and drug prediction of biomarkers in AS

To find out the regulatory mechanism of biomarkers in AS, miRNAs targeting biomarkers were predicted by miRDB database (http://mirdb.org) (score > 60). Next, long noncoding RNAs (lncRNAs)-targeting above miRNAs were found by starbase database (http://starbase.sysu.edu.cn/). After filtering and collecting, miRNA-lncRNA interaction pairs were obtained (clipExpNum > 20). Transcription factors (TFs) were also one of the main participants of regulatory network, comprehensive understanding of structure and dynamics of gene may complete by predicting TFs. This research predicted TFs by biomarkers in ChEA3 database (https://amp.pharm.mssm.edu/ChEA3) with *P* < 0.05. DSIGDB database (http://tanlab.ucdenver.edu/DSigDB) was carried out to predict target drugs by biomarkers for finding new targets and drugs of AS (*P* < 0.01). Ultimately, mRNA-miRNA-lncRNA, mRNA-TFs, and mRNA-drugs networks were all established using cytoscape software (version 3.10.2) [15].

### Immune infiltration analysis

To investigate the function of immunity in the development of AS, CIBERSORT algorithm was employed to assess the proportion of 22 immune cell types in AS and control samples. However, since the LM22 algorithm does not include major cells of vascular types, the xCell algorithm was additionally used to evaluate the proportion of 64 immune and stromal cell types in AS and control samples. The infiltration proportion plot was drafted by “ggplot2” package. Additionally, wilcoxon test was employed to assess the differential expression of cells between AS and control samples (*P* < 0.05). To explore the relationships between these cells and biomarkers, spearman correlation analysis was performed, and correlation plot was drafted by “ggpubr” package (version 0.6.0).

### scRNA-seq analysis

The scRNA-seq data in GSE159677 dataset was filtered using the “Seurat” package (version 5.1.0) [16] with following criteria: (1) cells with less than 200 genes (2), genes covered by fewer than 3 cells (3), cells with more than 20% proportion of mitochondrial genes (4), cells with genes ≤ 300 and ≥ 4,000 (5), genes with nCount ≤ 200 and ≥ 25,000. We standardized the data, and selected high-variable genes by FindVariableFeatures function in vst method. Following scaling scRNA-seq data by ScaleData function, *P* value of principal components (PCs) was calculated and compared by JackStrawPlot function. The significance degree of PCs was quantized, top 30 PCs which had significantly statistical discrepancy were selected for subsequent analysis (*P* < 0.05). Subsequently, unsupervised cluster analysis of cells was executed with the FindNeighbors and FindClusters functions from “Seurat” package to identify cell clusters, with cells were visualized by uniform manifold approximation and projection (UMAP) approach. Furthermore, cell subpopulations were annotated manually based on canonical marker genes reported in the literature and CellMarker database, rather than using automated reference-based methods [17]. The proportion of cell subpopulations in different samples was analyzed. Additionally, expression and distribution of biomarkers in cell subpopulations were analyzed, and compared by wilcoxon test (*P* < 0.05). To reveal the communication situation of cell subpopulations, ligand-receptor interaction was evaluated by “CellChat” package (version 1.6.1) [18]. A pseudotime analysis was then conducted using the “Monole” package (version 3.54.2) [18] to further examine the differentiation process of cell subpopulations with clustering cells and establishing cellular trajectories. The DiffialGeneTest Function was employed to investigate gene dynamics throughout cell differentiation. Trajectory was segmented according to trajectory nodes, expression of biomarkers was examined during whole differentiation stage.

### Animal study

A total of 4 male ApoE^−/−^ mice and 4 wild-type C57BL/6 mice (6–8 weeks old, weighing 18–24 g) were procured from GuangDong ZhaoQing Risemice Biotechnology Co., Ltd. All mice were acclimatized for one week in a specific-pathogen-free environment at the Laboratory Animal Center of GuangDong Pharmaceutical University prior to the commencement of the experiments. The wild-type C57BL/6 mice (Control group, *n* = 4) were fed a normal diet, while the ApoE^−/−^ mice (AS group, *n* = 4) were provided with a Western diet (Catalog No. 10141, Sinodiets JiLin, China) to induce atherosclerotic lesions. After 12 weeks, all mice were euthanized under isoflurane anesthesia, and carotid artery samples were collected for Western blot analyses. All animal procedures were conducted in accordance with protocols approved by the Animal Experiment Welfare Ethics Committee of GuangDong Pharmaceutical University (NO: gdpulacspf2022790).

### Oil Red O staining assay

After isolation of the entire aorta, the accumulation of lipids on the aortic vessel wall was observed by Oil Red O staining. Briefly, aortas were harvested from the aortic root to the iliac bifurcation and longitudinally opened using micro-scissors after removing external fat around aorta. The tissues were then fixed in 4% paraformaldehyde overnight at 4 ℃. Prior to staining, samples were washed three times with PBS and rinsed once with 60% isopropanol. Subsequently, they were stained with Oil Red O solution(G1015, Servicebio, China)for 5 min. After staining, nonspecific background staining was removed by washing three times with 60% isopropanol. Finally, the tissues were rinsed three times with PBS, flattened, and mounted on glass slides for imaging. Quantitative analysis was performed using Image-Pro Plus software to calculate the percentage of AS plaque area relative to the total intimal surface area of the aorta.

### Western blotting analysis

Western blotting was conducted as previously outlined [19]. Carotid artery samples were lysed with RIPA buffer (P0013B, Beyotime, China) supplemented with protease and phosphatase inhibitor cocktail (P1045, Beyotime, China), and protein concentrations were quantified using a BCA protein assay kit (P0010, Beyotime, China) following manufacturer’s instruction. Proteins were isolated via SDS-PAGE, subsequently transferred to PVDF membranes (Millipore, Bedford, MA, USA), and incubated with 5% (w/v) non-fat dry milk in Tris-buffered saline (TBS) containing 0.1% (v/v) Tween 20 for 1 h. Membranes were treated with the following diluted primary antibodies anti-XAF1(SAB2900401, Sigma), anti-NOD2(YP-mAb-18010,UpingBio, China), anti-NLRP3(YP-Ab-17813, UpingBio, China), anti-ANKRD1(YP-Ab-01588, UpingBio, China), anti-BDNF(YP-Ab-12472, UpingBio, China), anti-β actin (AF2815, Beyotime, China), and secondary antibodies including HRP-conjugated anti-mouse or anti-rabbit. Blots were visualized with the ECL detection system and quantified employing Image J software.

### Statistical analysis

All statistical analyses were conducted employing R software. The disparities were analyzed via Wilcoxon test. A statistically significant p value was less than 0.05.

## Results

### Identification of DEGs and AS-related module genes

**Fig. 2:**
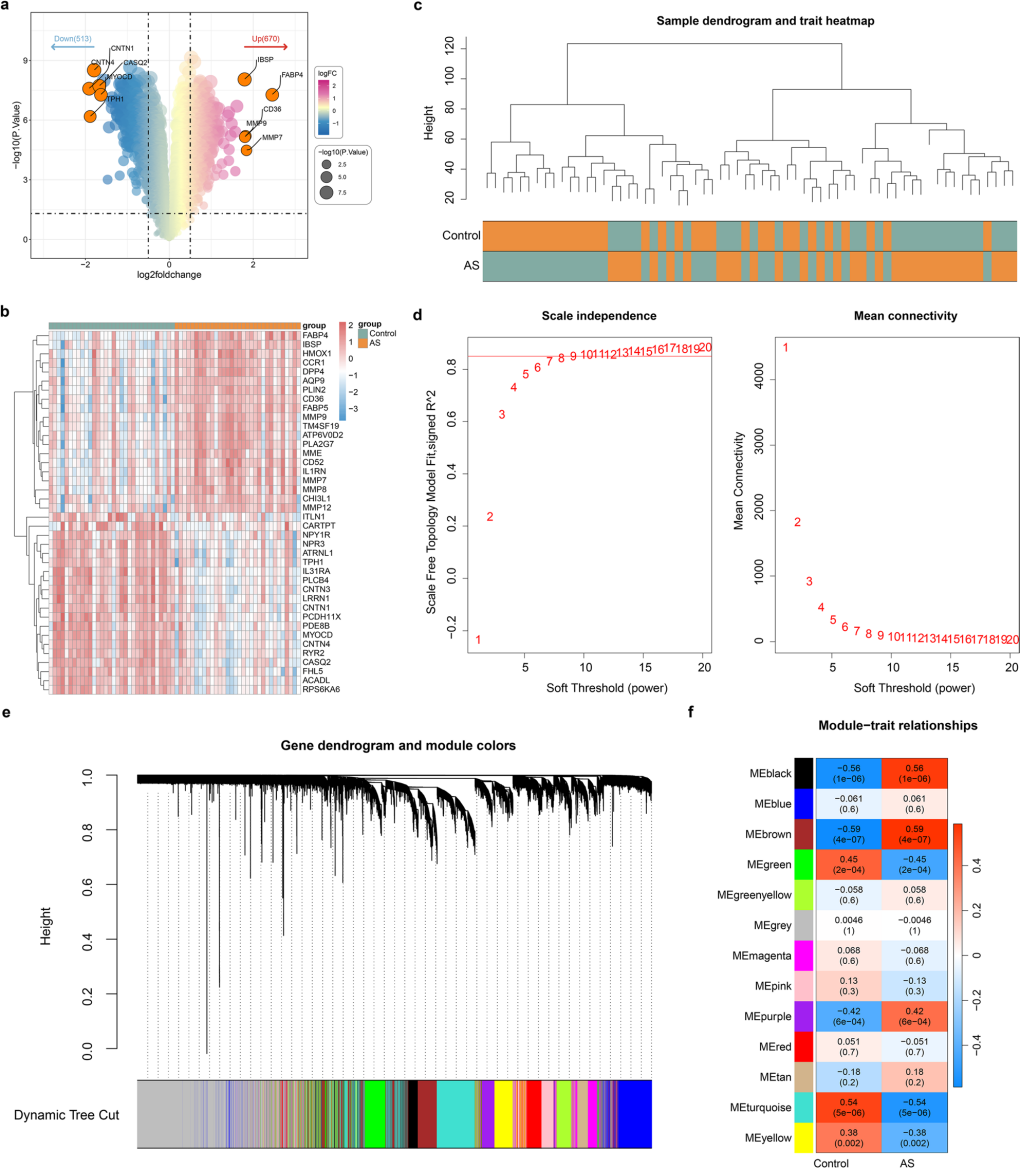
Selection of differentially expressed genes (DEGs) and atherosclerosis (AS)-related module genes (**a-b**) The volcano plot (**a**) and heat map (**b**) of DEGs between AS and control samples (**c**) Clustering dendrogram of samples in GSE43292 dataset (**d**) Selection of soft threshold (**e**) Clustering diagram of genes, various colors represents different modules (**f**) Correlation of 13 modules with 2 traits

Through differential expression analysis, 1,183 DEGs between AS and control samples were screened out, including 670 up-regulated and 513 down-regulated genes **(**Fig. 2a-b**)**. Cluster analysis was performed on all samples, and it was found that no outlier sample was present, so all samples were used for subsequent analysis **(**Fig. 2c**)**. The soft threshold in this research was set at 14 when R2 was closed to 0.85 and mean connectivity was approached 0 **(**Fig. 2d**)**. We further developed a hierarchical clustering tree, each branch representing genes with similar expression and biological functions. By setting 200 as the least number of genes, 13 modules were filtered out **(**Fig. 2e**)**. The correlation heatmap of selected modules and traits was displayed in Fig. 2F, and brown modules had the highest correlation with traits (|cor| = 0.59) were identified as important module. A total of 1,569 genes in important module were defined as AS-related module genes.

### Determination and analysis of DE-ERSRGs

**Fig. 3:**
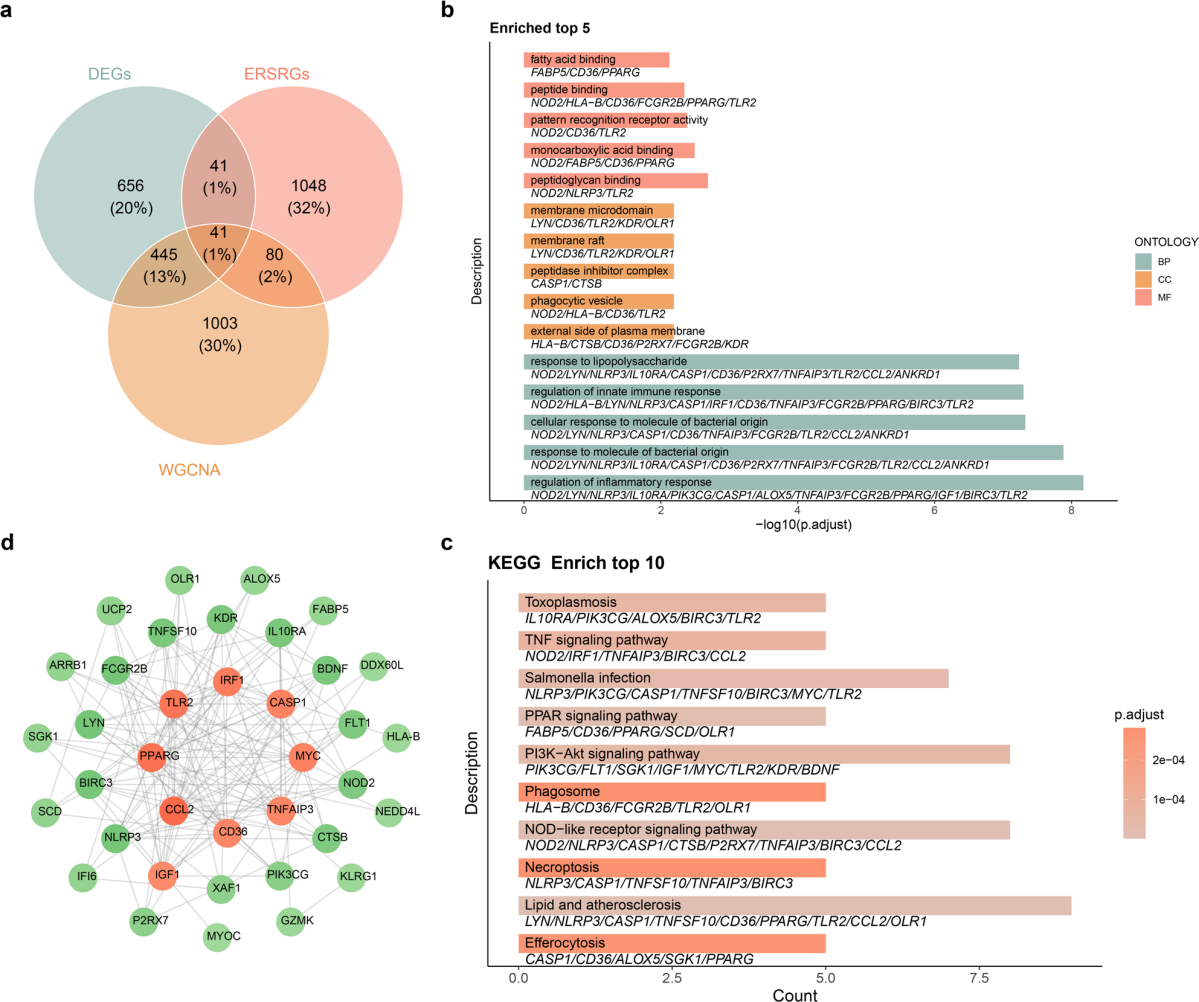
Determination and analyses of differentially expressed endoplasmic reticulum stress-related genes (DE-ERSRGs) (**a**) Venn diagram of DEGs, AS-related module genes and ERSRGs (**b-c**) Gene Ontology (GO) terms (**b**) and Kyoto Encyclopedia of Genes and Genomes (KEGG) pathways (**c**) enriched by DE-ERSRGs. BP, biological process; CC, cellular component; MF, molecular function (**d**) Protein-protein interaction (PPI) network of DE-ERSRGs. The orange circle represents more correlation with other genes than green circle

The intersection of DEGs, ERSRGs, and AS-related module genes yielded 41 genes, which were defined as DE-ERSRGs **(**Fig. 3a**)**. To investigate functional mechanism of DE-ERSRGs, GO and KEGG enrichment analyses were performed. We found that 690 GO terms and 29 KEGG pathways were enriched, including regulation of inflammatory response (GO-BP), external side of plasma membrane (GO-MF), peptidoglycan binding (GO-MF), and lipid and atherosclerosis (KEGG) **(**Fig. 3b-c**)**. Finally, the PPI network of DE-ERSRGs was constructed with 37 nodes and 151 interaction pairs. From this network, we found that PPARG, TLR2, and CCL2 had most correlation with other genes **(**Fig. 3d**)**.

### Activation of canonical ERS/UPR pathways in AS samples

To further evaluate whether canonical ERS pathways were activated in atherosclerosis, we examined the expression levels of key UPR markers in the GSE43292 dataset. We found that HSPA5, DDIT3, ATF4, EIF2AK3, ERN1, and ATF6 exhibited significant differential expression between atherosclerotic and control samples (Supplementary Fig. 1a).

In addition, GSEA based on established ERS/UPR-related gene sets demonstrated significant enrichment of canonical UPR pathways in atherosclerotic samples (Supplementary Fig. 1b-g). These results indicate that ERS activation in AS is characterized by coordinated engagement of canonical UPR signaling axes, supporting a pathway-level involvement of ERS beyond individual gene associations.

### Biomarkers in AS were selected by machine learning algorithms

**Fig. 4:**
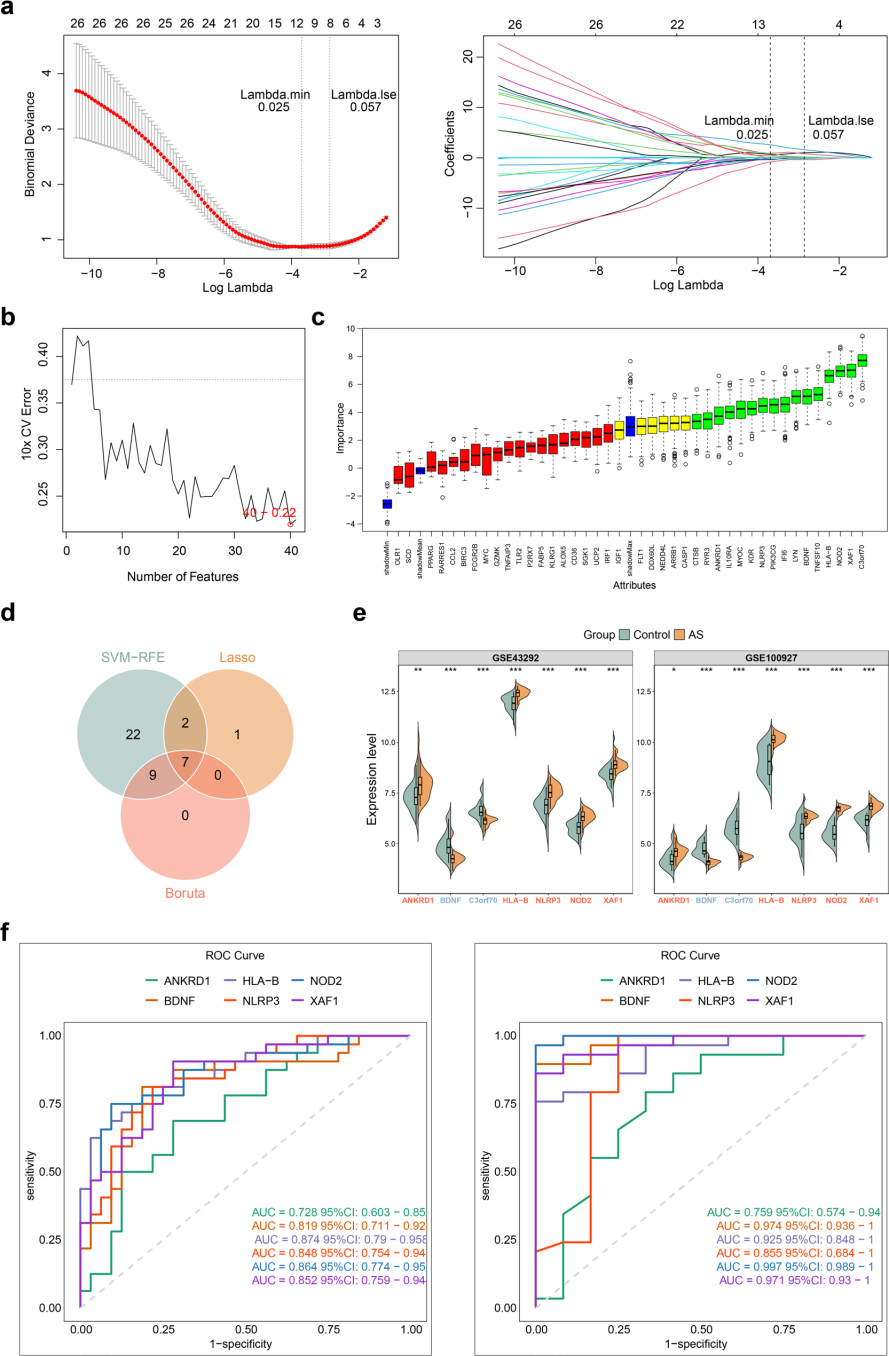
Identification of biomarkers for AS samples (**a**-**c**) Feature genes of least absolute shrinkage and selection operator (LASSO) (**a**), support vector machine-recursive feature elimination (SVM-RFE) (**b**), and Boruta (**c**) (**d**) Venn plot of feature genes derived from 3 machine learning algorithms (**e**) The expression of biomarkers between AS and control samples in GSE43292 and GSE100927 datasets (**f**) Receiver operating characteristic (ROC) curve of biomarkers in GSE43292 and GSE100927 datasets. AUC, area under the curve; 95%CI, 95% confidence interval

To further screen biomarkers in AS, machine learning algorithms were conducted. NOD2, HLA-B, NLRP3, XAF1, FLT1, ANKRD1, SCD, OLR1, C3orf70, and BDNF were selected as feature genes by LASSO at lambda.min = 0.025 **(**Fig. 4a**)**. When the error of SVM-RFE was the lowest (0.22), the number of feature genes was 40 **(**Fig. 4b**)**. A total of 16 feature genes were extracted by Boruta **(**Fig. 4c**)**. The interaction of above algorithms yielded 7 candidate biomarkers, including ANKRD1, BDNF, C3orf70, HLA-B, NLRP3, NOD2, and XAF1 **(**Fig. 4d**)**. The expression of candidate biomarkers was examined. We found that BDNF and C3orf70 had significantly higher expression in control samples, while remaining candidate biomarkers had converse results (*P* < 0.05) both in training and validation sets **(**Fig. 4e**)**. The AUC values of candidate biomarkers were all above 0.7 in both training and validation sets. However, C3orf70’s diagnostic performance did not generalize well in the validation cohort (Fig. 4f), indicating limited robustness and potential overfitting. Therefore, ANKRD1, BDNF, HLA-B, NLRP3, NOD2, and XAF1 were determined as final biomarkers for following analyses.

### Construction and verification of nomogram

**Fig. 5:**
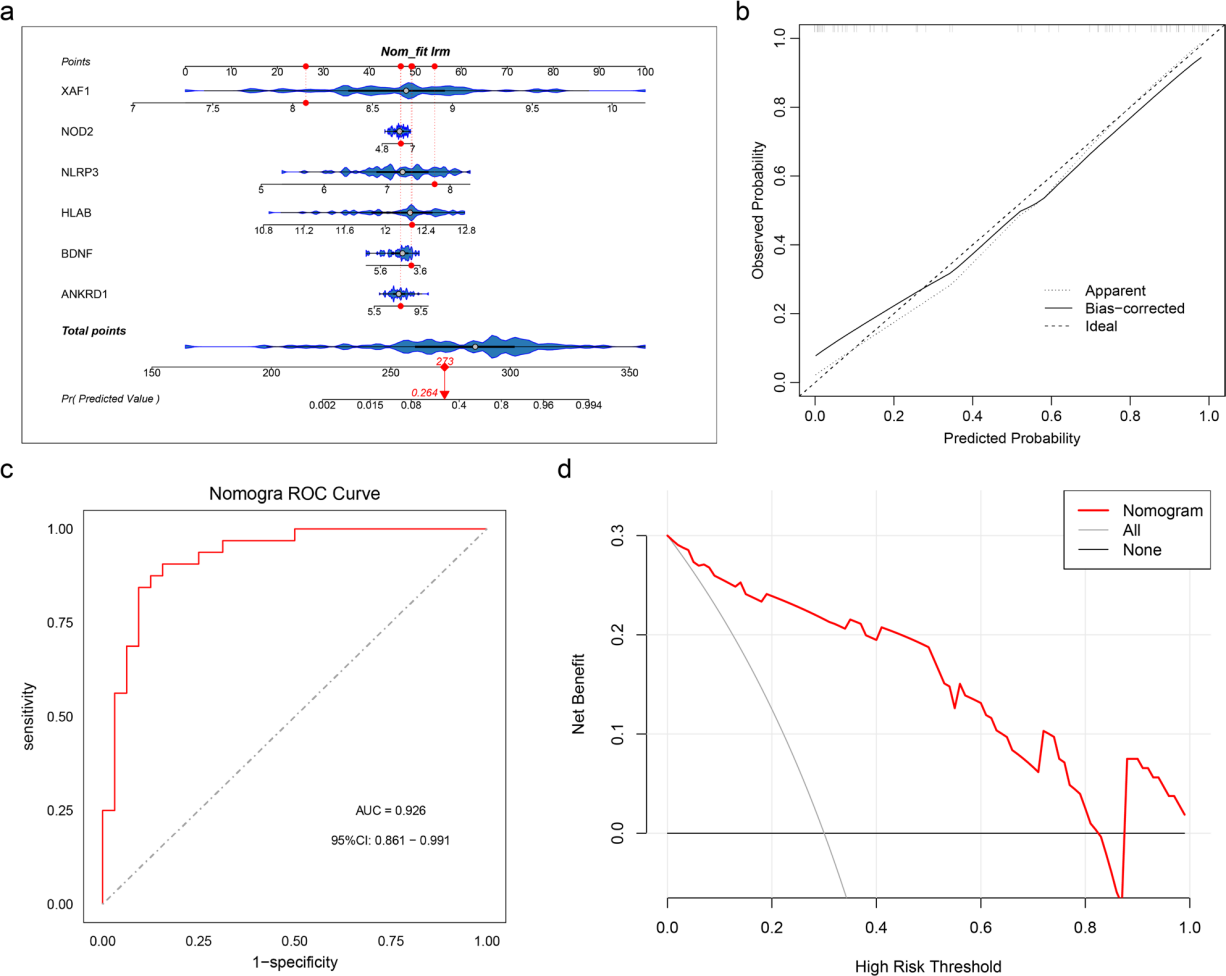
Construction of nomogram for predicting AS occurrence (GSE43292) (**a**) Construction of nomogram model (**b**) Calibration curve of nomogram (**c**) ROC curve of nomogram. AUC, area under the curve; 95%CI, 95% confidence interval (**d**) Decision curve analysis (DCA) curve

Using the GEO dataset (GSE43292), a nomogram was developed to assess the prediction capacity of biomarkers for the incidence of AS **(**Fig. 5a**)**. Calibration curve of the nomogram indicated that the prediction curve closely aligned with actual curve, demonstrating accurate predictive performance of nomogram **(**Fig. 5b**)**. AUC value of the ROC curve surpassed 0.7, signifying exceptional efficacy of nomogram **(**Fig. 5c**)**. In addition, decision curve analysis (DCA) demonstrated that the biomarker-based nomogram provided a higher net benefit across a range of threshold probabilities compared with treat-all and treat-none strategies, suggesting potential clinical usefulness **(**Fig. 5d**)**. Furthermore, to enhance the generalizability beyond the current validation cohort, we performed additional external validation using an independent GEO dataset (GSE111782). The results showed that the AUC was also greater than 0.7, and DCA indicated potential clinical value, which was consistent with the findings from the current cohort, suggesting the stability of our results (Supplementary Fig. 2a-c). All of these data demonstrated that the nomogram was an exceptional model for predicting the chance of AS. Compared with any single biomarker, the integrated nomogram demonstrated superior predictive performance, highlighting the added value of the multi-gene ERS-related model.

### Function analysis of biomarkers

**Fig. 6:**
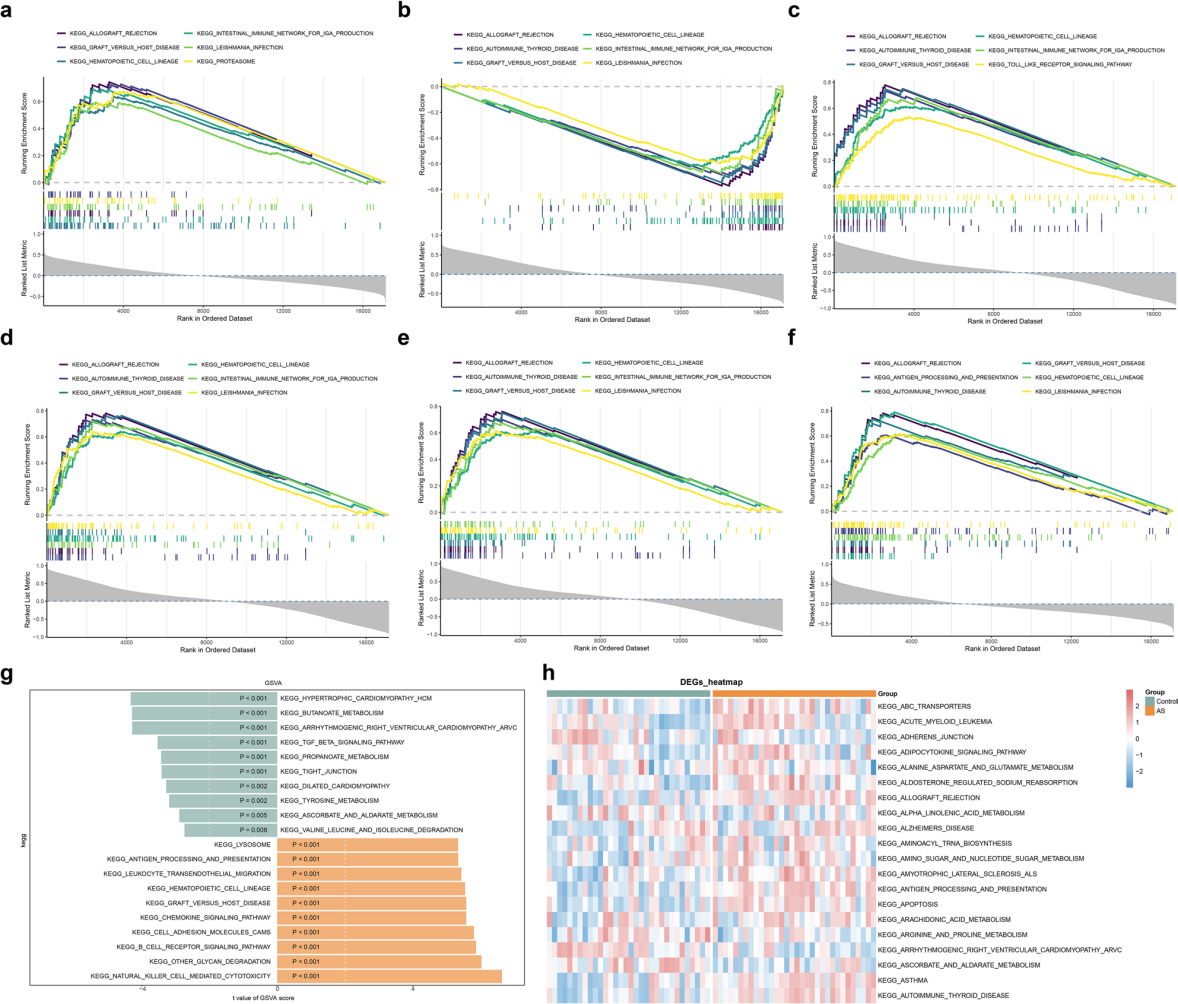
Gene set enrichment analysis (GSEA) and gene set variation analysis (GSVA) (**a**-**f**) GSEA of ANKRD1 (**a**), BDNF (**b**), HLA-B (**c**), NLRP3 (**d**), NOD2 (**e**), and XAF1 (**f**) (**g**) The differential pathways of GSVA in AS and control samples (**h**) Heatmap of GSVA

To clarify potential functions of each biomarker, single gene GSEA-KEGG pathway analysis was performed. Results showed that 43, 53, 50, 47, 51, and 33 pathways were respectively enriched by ANKRD1, BDNF, HLA-B, NLRP3, NOD2, and XAF1. BDNF was negatively correlated with hematopoietic cell lineage, allograft rejection, and graft versus host disease, while other biomarkers were positively related with these common pathways **(**Fig. 6a-f**)**. As a comprehensive analysis, we found above pathways were associated with immune, this suggested that biomarkers may act on AS through the regulation of the immune system.

Additionally, GSVA was applied to find the KEGG pathways that correlated with the development of AS. The results showed that 95 pathways considerably different between AS and control samples. Among these pathways, 21 were inhibited in AS samples, including hypertrophic cardiomyopathy hcm and butanoate metabolism, while 74 pathways were activated in AS samples, containing natural killer cell mediated cytotoxicity, other glycan degradation, and B cell receptor signaling pathway **(**Fig. 6g-h**)**.

### Construction of interaction network of biomarkers

**Fig. 7:**
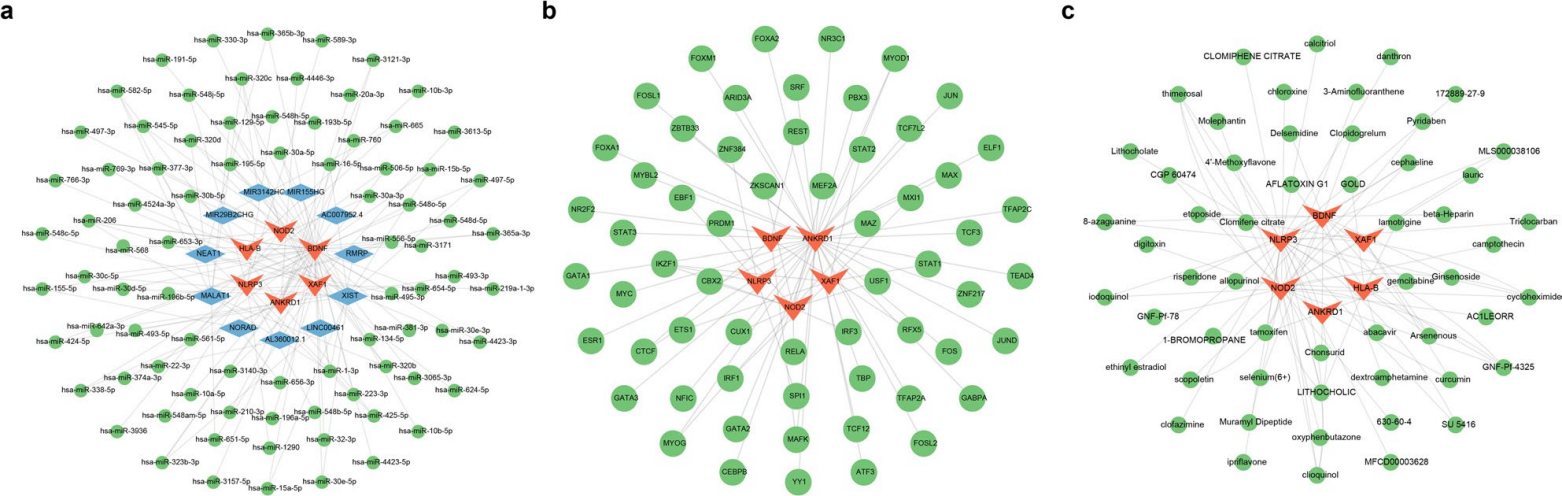
Regulatory network of biomarkers (**a**) Construction of mRNA-microRNA (miRNA)-long noncoding RNA (lncRNA) regulatory network. Orange denotes mRNA, green represents miRNA, blue represents lncRNA (**b**) Regulatory network of biomarkers and transcription factors (TFs). Orange represents biomarkers, green denotes TFs (**c**) Potential drugs of biomarkers in AS samples. Orange represents biomarkers, green denotes drugs

Through miRDB and starbase database, a total of 83 miRNAs and 41 miRNAs-lncRNAs interaction pairs were predicted. After merging and removing duplication, a mRNA-miRNA-lncRNA regulatory network was constructed with 100 nodes and 127 relation pairs (6 mRNAs, 83 miRNAs, and 11 lncRNAs) **(**Fig. 7a**)**. These regulatory relationships included BDNF-hsa-miR-1-3p-MALAT1, NLRP3-hsa-miR-22-3p-AL360012.1, and ANKRD1-hsa-miR-425-5p-NEAT1. Through ChEA3 database, 39 TFs regulated ANKRD1, 4 TFs regulated BDNF, 4 TFs regulated NLRP3, 12 TFs regulated NOD2, 7 TFs regulated XAF1 were obtained. After integrating, a mRNA-TF regulatory network was created with 65 nodes, and 66 relation pairs **(**Fig. 7b**)**. IKZF1 regulated XAF1, NOD2, and NLRP3, while a total of 4 TFs regulated NLRP3, namely IKZF1, SPI1, ZNF384, EBF1. Totally 39 TFs regulated ANKRD1, including TEAD4, NR3C1, FOSL1, and etc. Moreover, 55 types of potential drugs for AS were predicted by biomarkers, and a mRNA-drug network was established with 61 nodes and 84 edges. Among these drugs, 2 drugs interacted with ANKRD1 (tamoxifen and gemcitabine), 15 drugs interacted with BDNF, 7 drugs interacted with HLA-B, 30 drugs interacted with NLRP3, 27 drugs interacted with NOD2, while 3 drugs interacted with XAF1 (arsenenous, etoposide, and curcumin). Thimerosal, GNF-Pf-4325, cycloheximide, MLS000038106, iodoquinol, cephaeline, and clioquinol were predicted by NLRP3 and NOD2, synchronously. Tamoxifen, cephaeline, and 8-azaguanine were predicted by BDNF and NLRP3 **(**Fig. 7c**)**.

### The condition of immune infiltration in AS samples

**Fig. 8:**
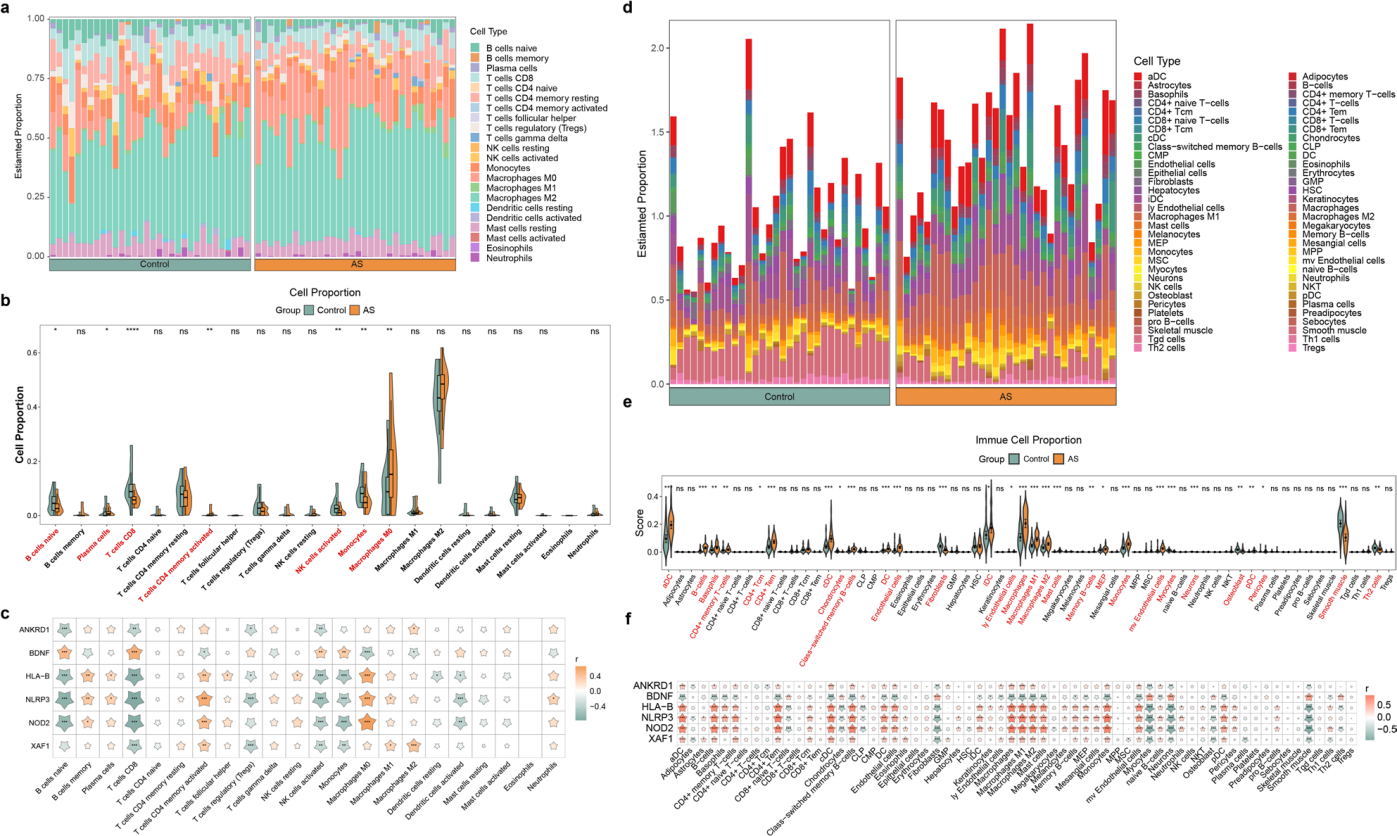
Immune infiltration analysis in AS and control samples (**a**) Relative abundance of 22 infiltrating immune cell types in AS and control samples (**b**) Differences in 22 immune cell types between AS and control samples (**c**) Correlations between immune cell types and biomarkers (**d**) xCell-based enrichment scores of significantly different cell types in AS and control samples (**e**) Differences in xCell-identified cell types between AS and control samples (**f**) Correlations between xCell-derived cell types and biomarkers. ns: not significance * *P* < 0.05, ** *P* < 0.01, *** *P* < 0.001, **** *P* < 0.0001

Previous studies have shown that immunity played a critical roles in AS, so CIBERSORT algorithm was employed in this study to analyze immune infiltration of 22 immune cells in AS and normal samples **(**Fig. 8a**)**. Difference of immune cells was estimated, and we found that plasma cells, activated memory CD4 T cells, and M0 macrophages had obviously higher expression in AS samples, while naive B cells, CD8 T cells, activated NK cells, and monocytes had significantly lower expression in AS samples **(**Fig. 8b**)**. Furthermore, correlation analysis showed that NOD2 had the strongest positive relation with M0 macrophage (cor = 0.658, *P* < 0.05), while NLRP3 had the strongest negative correlation with CD8 T cells (cor = -0.771, *P* < 0.05) **(**Fig. 8c, Supplementary Fig. 3a-f).

Given that the LM22 signature is optimized for immune cells and does not explicitly include major vascular cell types, we further applied the xCell algorithm, which incorporates both immune and non-immune cell signatures, to provide a complementary and vascular-aware assessment of tissue composition. Using xCell, 29 cell types exhibited significant differences between AS and control samples (Fig. 8d-e). Consistent with immune involvement, NLRP3 showed the strongest positive correlation with Monocytes (*r* = 0.927, *P* < 0.01), whereas NOD2 displayed the strongest negative correlation with Smooth muscle cells (*r* = -0.912, *P* < 0.01) (Fig. 8f). These results support that the observed immune-related associations are robust and remain evident when using a deconvolution approach that accounts for vascular cell components.

### Special cell subpopulations were selected

**Fig. 9:**
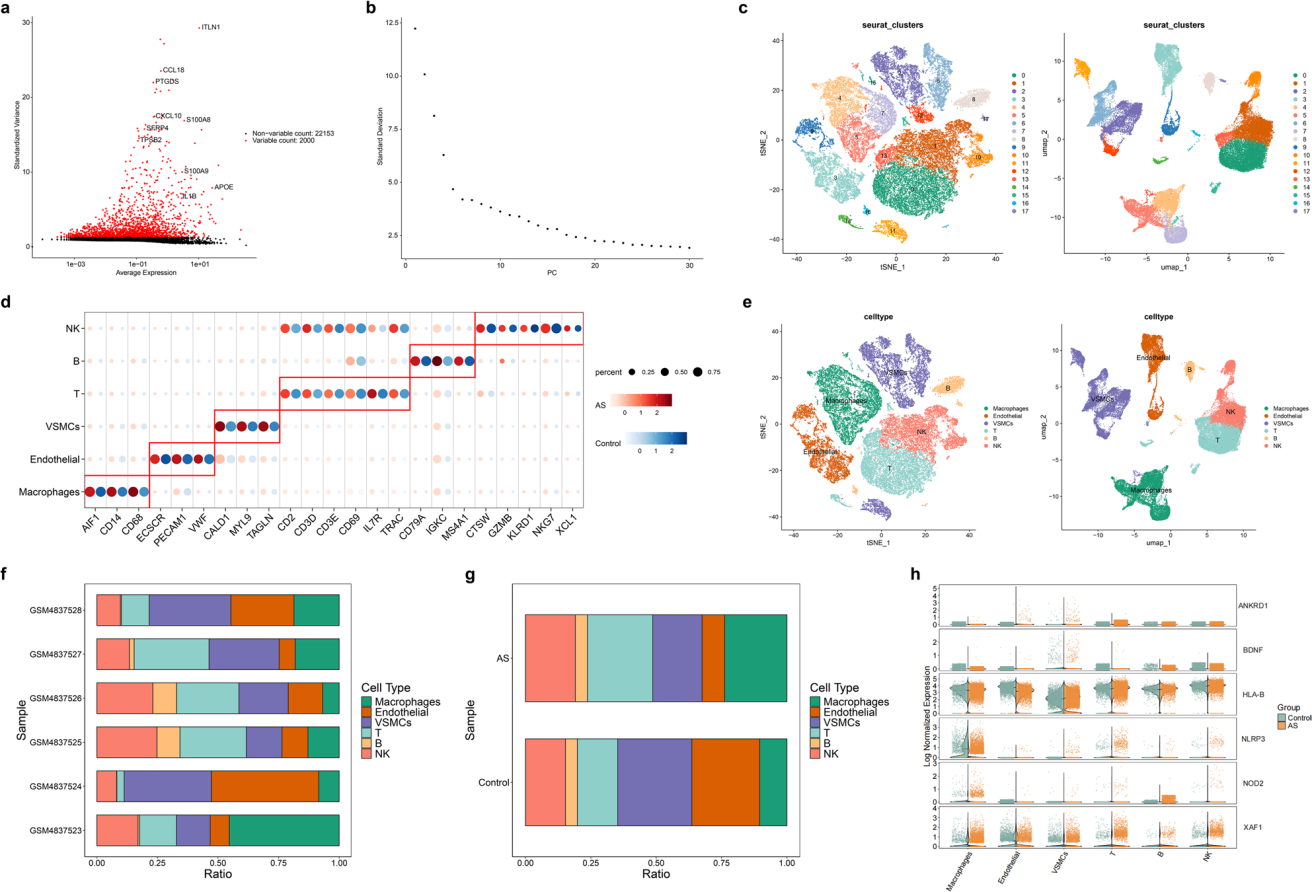
Single cell RNA sequencing (scRNA-seq) analysis (**a**) Top 10 genes of high variable genes (**b**) Top 30 principal components (PCs) of p value (**c**) Identification of 18 cell clusters (**d**) The marker genes of each cell type (**e**) Annotation of cell subpopulations (**f**) The proportion of cell subpopulation in different samples (**g**) The proportion of cell subpopulations between AS and control samples (**h**) The expression of biomarkers in cell subpopulations between AS and control samples

To explore the cell characteristic of AS samples in single cell level, the data in GSE159677 dataset was screened, and 48,196 cells were remained for subsequent analysis (Supplementary Fig. 4). Then, 2,000 high-variable genes were displayed, and top 30 PCs from PCA with statistical significance were screened out for following analysis **(**Fig. 9a-b**)**. Furthermore, we identified 18 cell clusters **(**Fig. 9c**)**, among which 6 cell subpopulations including NK cells, B cells, T cells, vascular smooth muscle cells (VSMCs), endothelial, and macrophages were next annotated by marker genes of each cell types **(**Fig. 9d-e, Supplementary Table 1). Through proportion analysis of cell subpopulations in AS and control samples, distinct distribution patterns were observed, with higher proportions of NK cells, T cells and macrophages in AS samples, whereas VSMCs and endothelial cells were relatively enriched in control samples, while VSMCs and endothelial were more in control samples **(**Fig. 9f-g**)**. The expression analysis demonstrated that ANKRD1, BDNF, HLA-B, and NLRP3 displayed cell-type associated expression differences between AS and control samples in VSMCs. ANKRD1, HLA-B, and XAF1 had obvious difference in endothelial, while HLA-B, NLRP3, and XAF1 had significantly differential expression in T cells **(**Fig. 9h, Supplementary Fig. 4a-g).

**Fig. 10:**
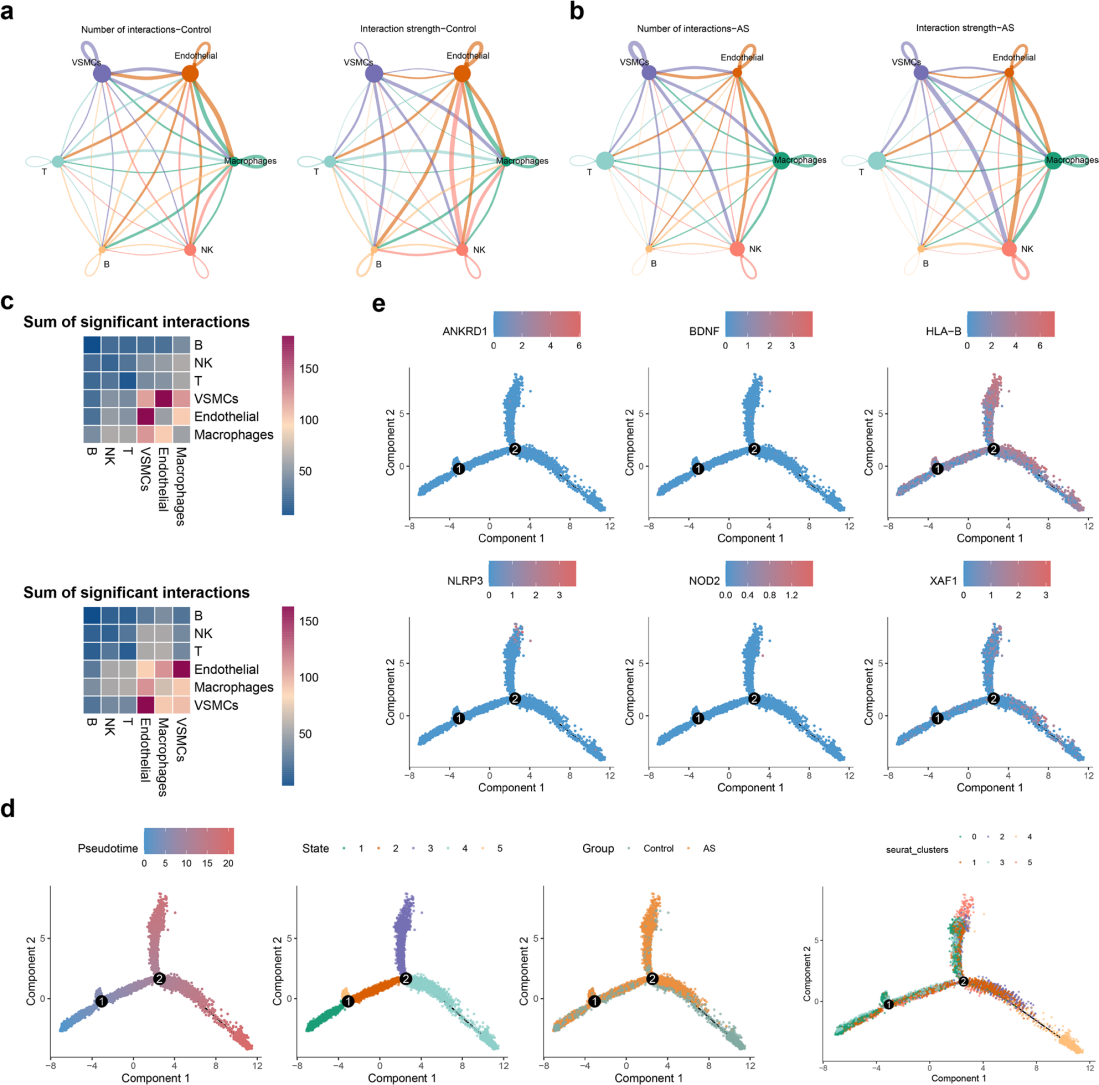
Cell communication and pseudo-time trajectory analysis (**a**-**b**) Communication of cell subpopulations in control (**a**) and AS samples (**b**) (**c**) Ligand-receptor-mediated communication between VSMCs and other cell types (Top: Control; Bottom: AS) (**d**) Pseudo-time trajectory analysis of vascular smooth muscle cells (VSMCs) (**e**) The expression of biomarkers during VSMCs differentiation

Furthermore, communication of cell subpopulations was also explored by cellchat, we found communication number and strength of cells were changes in diverse samples. For example, VSMCs exhibited enhanced inferred interaction patterns with other cell subpopulations in AS samples **(**Fig. 10a-c, Supplementary Fig. 5a-b). Phenotypic alteration of VSMCs was pivotal in the pathological progression of AS, so pseudo-time trajectory analysis of VSMCs was performed. The results indicated that HLA-B exhibited higher expression in earlier/intermediate pseudotime states, whereas XAF1 expression increased toward later pseudotime states **(**Fig. 10d-e**)**.

### Validation of atherosclerotic plaque formation in mouse aortas

**Fig. 11:**
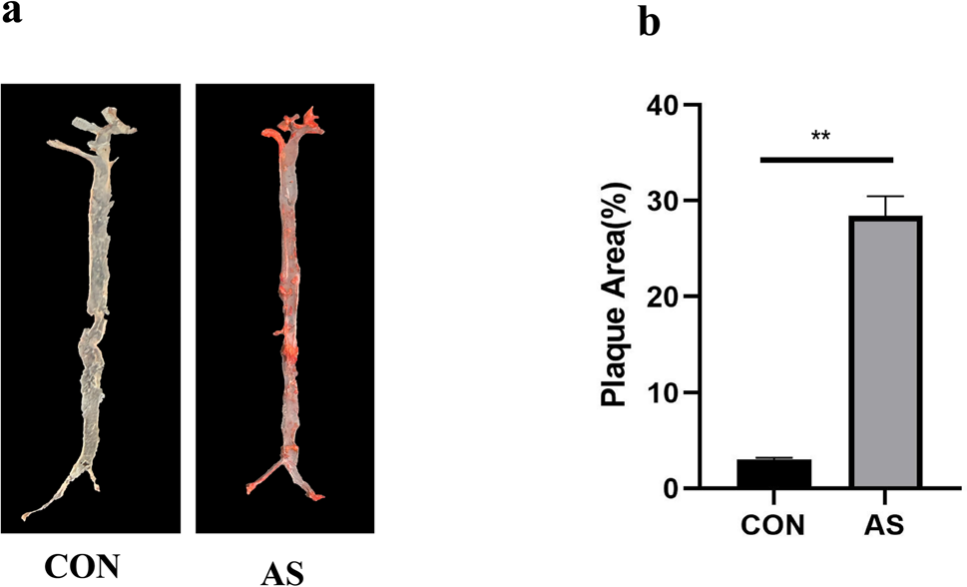
Representative images and quantification of oil red O staining in mouse aortas Representative images of oil red O staining of aortas from mice fed with a normal diet (CON) or a high-fat diet (AS). Red staining indicates lipid-rich atherosclerotic plaques in mouse aortas (**a**). Quantitative analysis of the plaque area from the two groups (**b**). Data are presented as mean ± SD. (*n* = 4,**P < 0.01)

To confirm the successful establishment of the AS mouse model, atherosclerotic plaques in mouse aortas was assessed via oil red O staining. Representative images of aortas from mice fed a normal diet (CON) or a high-fat diet (AS) are shown in Fig. 11a). Extensive red-stained lipid-rich plaques were visibly present in the AS group, whereas aortas from the CON group remained largely clear. Quantitative analysis further confirmed a significant increase in the percentage of plaque area relative to the total aortic surface area in AS group compared to CON group (Fig. 11b).

### Detection of the biomarkers expression in carotid artery by western blot

**Fig. 12:**
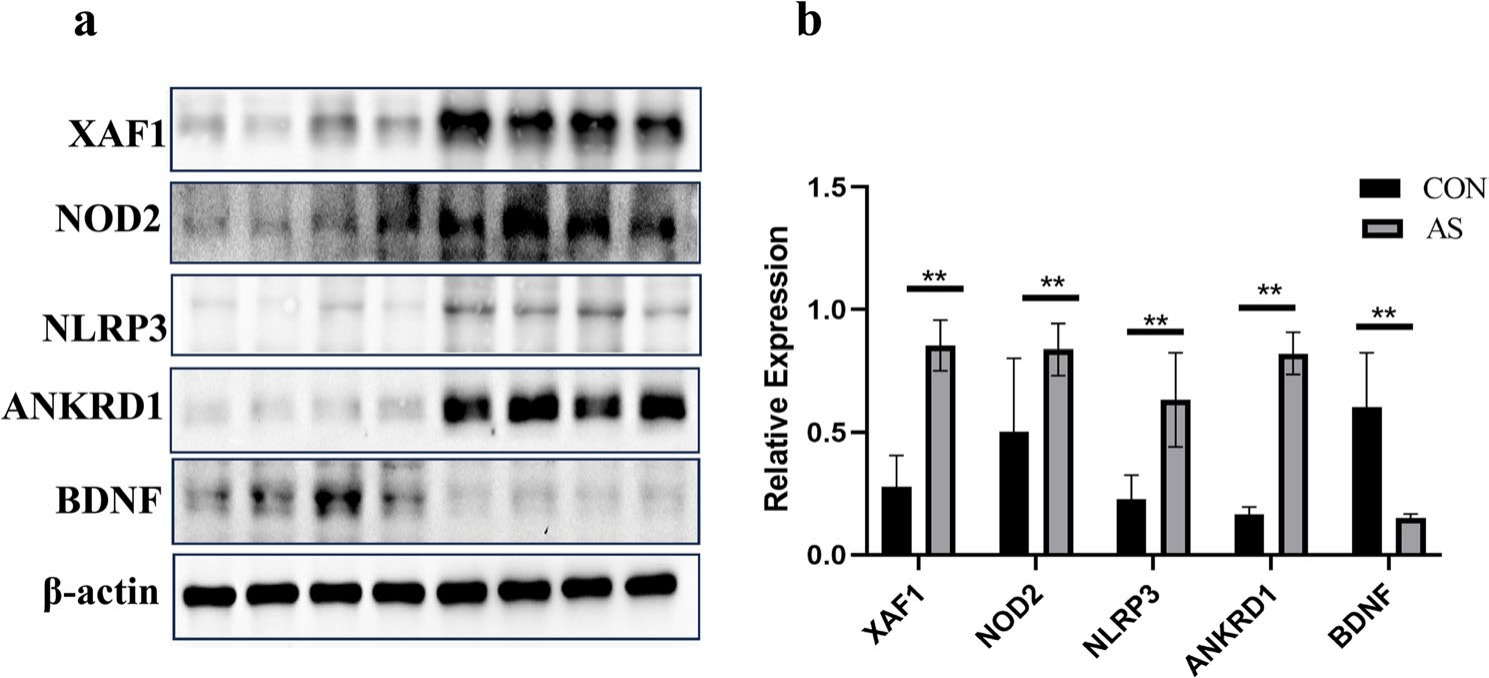
The expression of XAF1, NOD2, NLRP3, ANKRD1 and BDNF at the protein level in mouse carotid artery **(a)** Protein levels were detected by Western Blot in wild-type C57BL/6 mice (Control group, *n* = 4) were fed a normal diet, while the ApoE^−/−^ mice (AS group, *n* = 4) were provided with a Western diet to induce atherosclerotic lesions. **(b)** The relative expression of the protein levels in AS mouse carotid artery.(*n* = 4,**P < 0.01 )

The biomarkers of XAF1, NOD2, NLRP3, ANKRD1 and BDNF were detected by western blot. As a result, we explored for the first time the link between XAF1 and AS. XAF1 was highly expressed in the AS group compared to control group at the protein level (*P* < 0.01) **(**Fig. 12**)**. Consistent with our bioinformatic prediction, the expression levels of the other candidate proteins were also validated in this model. Western blot analysis confirmed that the expression of NOD2, NLRP3, and ANKRD1 were significantly upregulated in the AS group, whereas the expression of BDNF was downregulated compared to the CON group (Fig. 12). Meanwhile, the biomarkers for AS have been investigated previously (ANKRD1, NLRP3, NOD2, BDNF) [20–23]. The human HLA-B gene corresponds to the H-2k, H-2D, and I-Ab genes in C57BL/6 mice. Unfortunately, we were unable to get the commercialized H-2k, H-2D and I-Ab antibodies for Western blot analysis.

## Discussion

AS remains a leading cause of CVDs worldwide, significantly contributing to both morbidity and mortality rates [24]. The progression of AS is intricately associated with ERS. Previous studies have indicated that the ERS markers such as ATF6, XBP1, and IRE1 are activated in endothelial cells (ECs) in regions of the aorta susceptible to atherosclerotic [25]. Pathways associated to ERS in AS have also been examined. Liwei Hang et al.. demonstrated that oxidized low-density lipoprotein (ox-LDL) triggers inflammatory alterations in ECs through inflammasome activation, mediated by apoptosis signal-regulating kinase 1 (ASK1)/NOD-, LRR-, and pyrin domain-containing protein 3 (NLRP3), resulting in EC damage via ERS [26].

Notably, under chronic stress conditions, ERS is known to shift from an initially adaptive unfolded protein response toward maladaptive inflammation and apoptosis [8, 27], thereby contributing to disease progression and providing a mechanistic context for interpreting ERS-related molecular alterations in AS. These findings have deepened our understanding of ERS in AS. However, comprehensive identification of biomarkers related to ERS in AS remains limited. Importantly, although several inflammation-related genes have been individually reported in AS, most previous studies did not explicitly constrain biomarker discovery to endoplasmic reticulum stress or integrate multi-level validation strategies. Here, we addressed this gap by establishing an ERS-constrained biomarker discovery framework combined with machine learning, external validation, and single-cell resolution analyses.

Rather than re-evaluating single genes, our results highlight the collective diagnostic and mechanistic relevance of an ERS-related gene signature in AS. In the present study, we identified six ERS-associated biomarkers (ANKRD1, BDNF, HLA-B, NLRP3, NOD2, and XAF1), which exhibit strong correlations with AS pathogenesis. The apoptosis of VSMCs plays a significant role in the progression of AS, particularly in vascular remodeling and plaque rupture [28]. Recent research found that ERS inducer tunicamycin reduces cell viability and increases the number of TUNEL-positive cells in VSMCs. Additionally, ankyrin repeat domain1 (ANKRD1) expression was markedly downregulated following tunicamycin stimulation in VSMCs, highlighting its potential as a therapeutic target for AS [29]. Similarly, Brain-Derived Neurotrophic Factor (BDNF) is traditionally known for its pivotal role in regulating neuronal survival and the plasticity of neurons. Additionally, some studies have demonstrated that BDNF is involved in the regulation of endothelial function and the progression of AS via chronic unpredictable stress [23, 30]. Human leukocyte antigen B (HLA-B), a major histocompatibility complex (MHC) gene, exhibits significant genetic associations with AS susceptibility. Lihi Eder et al. reported that differential alleles are associated with the severity of AS. This association is likely mediated, at least in part, by systemic inflammation and a higher cumulative burden of disease activity over time [31]. Furthermore, the NLRP3 inflammasome is highly expressed in both innate immune cells and non-immune cells implicated in the pathogenesis of atherosclerotic cardiovascular disease [32]. Studies have demonstrated that inhibiting NLRP3 may have protective effects against inflammatory damage in AS [33]. Nucleotide-binding oligomerization domain 2 (NOD2) was initially regarded as a susceptibility gene for Crohn’s disease. Recent studies have shown that it is not only a bacterial sensing receptor but also plays a significant role in regulating immune responses [34]. AS is a chronic inflammatory disease, and its pathological progression is closely related to immune balance, NOD2 may play a key role in this process [35]. Atherosclerotic plaque rupture and the incidence of myocardial infarction occur more often in the morning compared to other times of the day. XAF1 is believed to play an important role in the induction of apoptosis. For example, XAF1 can dramatically sensitize cancer cells to apoptotic triggers, such as TNF-related apoptosis-inducing ligand (TRAIL), etoposide, 5-fluorouracil [36, 37].In this study, we observed increased XAF1 protein expression in the carotid arteries of AS mice. Given that apoptosis has been implicated in plaque vulnerability and thrombosis [38], and that prolonged endoplasmic reticulum stress (ERS) can activate apoptosis-related pathways, the upregulation of XAF1 in AS lesions may reflect ERS-associated apoptotic signaling during plaque progression. This finding suggests a potential association between XAF1 upregulation and lesion progression, which warrants further functional validation. Meanwhile, Schober A et al. have found that apoptosis within atherosclerotic lesions showed diurnal oscillation, peaking in the early morning, in phase with XAF1 expression [39]. These observations raise the possibility that XAF1 may be involved in pathways related to plaque vulnerability. However, whether XAF1 directly contributes to plaque instability or can be therapeutically targeted requires dedicated mechanistic studies. Lastly, all these findings underscore the involvement of biomarkers in key processes, including cell apoptosis, endothelial dysfunction, inflammation, and immune regulation, suggests their potential as novel therapeutic targets and diagnostic markers for AS. Further research into these biomarkers could provide valuable insights into the development of more effective treatments for AS.

Thus, a nomogram was exhibited based on biomarkers for AS, which demonstrates potential clinical applicability, as previously highlighted in studies [40]. The development of a nomgram is essential for fully understanding the predictive capabilities of all biomarkers for the occurrence of AS. Our results revealed that the nomogram exhibited excellent predictive performance, with AUC = 0.926, which indicated that nomogram was reliable and valuable tool for assessing AS risk. This finding underscores the added predictive value of integrating multiple ERS-related biomarkers into a single model, rather than relying on individual markers alone. By integrating multiple biomarkers, this nomogram enhanced our ability to predict AS onset with greater accuracy, making it a crucial asset in clinical practice for personalized risk stratification and therapeutic decision-making.

The intricate interplays among transcriptional regulation and immune profiles in AS are underscored by construction of regulatory networks and immune infiltration analysis. In this study, immune infiltration was primarily assessed using CIBERSORT with the LM22 signature to characterize relative changes in immune-lineage cells associated with AS. Given that LM22 does not explicitly include major vascular cell types, we additionally performed complementary analyses using the xCell algorithm, which incorporates both immune and non-immune (including vascular) cell signatures, and observed consistent immune-related patterns.

Results show that NLRP3, NOD2, and XAF1 are regulated by IKZF1. IKZF1 (IKAROS family zinc finger 1) is a transcription factor vital to the immune system. Mutations in IKZF1 affecting both B and T cells have been documented in human hematologic malignancies and primary immunodeficiencies [41, 42]. Consistent with above findings, our analyses also discover significant alterations in immune cells, such as increased plasma cells, activated memory CD4 + T cells, and M0 macrophages, alongside a decrease in naïve B cells, CD8 + T cells, activated NK cells, and monocytes. Initial studies have found that infiltrating CD4 + T cells influences in AS by interacting with oxidized low-density lipoprotein and other antigens. This interaction promotes a pro-inflammatory environment that accelerates plaque formation and vascular damage [43]. Otherwise, Xu et al. discovered that interferon-γ derived from CD8 + T cells promotes macrophage activation, thereby inducing AS [44]. As mentioned earlier, various T-cell subsets, including CD4 + and CD8 + T cells, NK T cells, and follicular helper T cells, are present within human atherosclerotic lesions, suggesting that these subsets contribute to a more intricate immune landscape, where their interactions with other immune cells and endothelial cells promote a sustained inflammatory response within the arterial wall [45–48]. These findings all underscrore the importance of immune cell infiltration in the development and complications of AS.

Single-cell sequencing technology and analytical tools can provide new insights into AS progression. We further utilized the advance single-cell sequencing technology identified six cell subpopulations including NK cells, B cells, T cells, VSMCs, endothelial, and macrophages. Compared to control samples, the proportion of VSMCs in AS samples was decreased. The historical perspective on VSMCs in AS is that “aberrant” proliferation of VSMCs contributes to plaque formation, while VSMCs in advanced plaques are entirely beneficial, such as blocking rupture of the fibrous cap [49]. Furthermore, communication of cell subpopulations was also explored by cellchat, we found VSMCs had more and stronger correlation with other cell subpopulations in AS samples. The observed prominence of VSMC-mediated intercellular communication likely reflects their remarkable phenotypic plasticity. Wirka RC et al. and Shankman LS et al.. demonstrated that VSMCs lose their contractile markers during the process of AS and instead display multiple phenotypes similar to Myofibroblast-like VSMCs, Macrophage-like VSMCs etc [50, 51]. Notably, by integrating single-cell communication and pseudotime analyses, our study provides cell-type specific and state-resolved evidence, which cannot be inferred from bulk transcriptomic analyses alone.

Our study has successfully established a connection between ERS with AS via six biomarkers, offering promising new diagnostic targets for AS. These findings deepen our understanding of the molecular mechanisms in AS and pave the way for more precise diagnostic tools.

However, our study also had several limitations. First, the sample size was relatively small, which may affect the generalizability and clinical applicability of the identified biomarkers. In addition, although public transcriptomic datasets were used for both training and validation, external validation across larger and more diverse independent cohorts remains limited, which may influence the robustness of certain findings. Moreover, while training and validation were conducted in independent cohorts, explicit cross-platform harmonization procedures were not applied, which may limit strict platform-invariant inference. Expanding the sample size in future studies will be essential to improve the robustness and reliability of these findings.

Second, the relationships between the identified biomarkers and ERS are currently inferred mainly from bioinformatics analyses and existing literature, and direct experimental validation under ERS perturbation conditions is required in future studies. Although our study primarily relied on bioinformatics approaches, in vitro, in vivo, and clinical experiments were largely lacking, which further limits the strength of causal inference. In addition, the cross-sectional nature of the available public GEO datasets, together with limited sample size and incomplete clinical covariate information, precluded robust mediation-style analyses to formally quantify the extent to which ERS signatures mediate the association between biomarkers and disease status.

Third, the proposed functional roles of XAF1 in VSMC apoptosis and plaque instability are based mainly on expression-level evidence and previous reports, and therefore should be interpreted with caution until confirmed by dedicated loss- and gain-of-function experiments as well as independent clinical cohorts.

Fourth, detailed information on patient therapies and comorbidities was not consistently available in the public GEO metadata, precluding formal covariate adjustment. Although the paired design of the training cohort partially mitigates subject-level confounding, residual confounding cannot be excluded. Therefore, the identified biomarkers should be interpreted as association signals requiring validation in well-phenotyped cohorts. In addition, the lack of standardized clinical risk factors in the public GEO datasets precluded formal comparison against clinical-only models and calculation of incremental metrics such as NRI and IDI. Meanwhile, the current findings are limited by validation in tissue samples only and lacking of spatial and cell-type-specific localization data for the validated biomarkers within human plaques. Future work will focus on assessing these biomarkers in blood to translate them into clinically feasible liquid biopsy tests and employing immunohistochemistry or in situ hybridization are warranted to define their precise anatomical context. Further research into the underlying mechanisms of these biomarkers is necessary to better understand their potential for clinical application and therapeutic intervention.

## Conclusions

In conclusion, this study employ various bioinformatics methods to identify six ERS-related biomarkers (ANKRD1, BDNF, HLA-B, NLRP3, NOD2 and XAF1) that are associated with atherosclerosis. Our findings reveal that these biomarkers are closely associated with immune system processes involved in the development and progression of AS. Although the identified biomarkers and the proposed nomogram demonstrate potential utility for risk stratification, further experimental validation and studies in clinically well-phenotyped cohorts are required to clarify their functional roles and to establish their translational and clinical relevance in AS.

## Supplementary Information

Supplementary Material 1. Activation of canonical Endoplasmic Reticulum Stress (ERS)/Unfolded Protein Response (UPR) pathways in AS samples (a) Differential expression of key UPR markers between atherosclerotic and control samples (b-g) GSEA analysis of ATF4 (b), ATF6 (c), DDIT3 (d), EIF2AK3 (e), ERN1 (f), and HSPA5 (g).

Supplementary Material 2. Construction of nomogram for predicting AS occurrence (GSE111782) (a) Construction of nomogram model (b) ROC curve of nomogram. AUC, area under the curve; 95%CI, 95% confidence interval (c) Decision curve analysis (DCA) curve.

Supplementary Material 3. The expression of biomarkers in immune cells (a-f) The correlation lollipop plot of immune cells with ANKRD1 (a), BDNF (b), HLA-B (c), NLRP3 (d), NOD2 (e), and XAF1 (f).

Supplementary Material 4. Single-cell RNA sequencing (scRNA-seq) analysis in cell subpopulations (a) Quality control of data in GSE159677 dataset. (b-g) The expression of ANKRD1 (b), BDNF (c), HLA-B (d), NLRP3 (e), NOD2 (f), and XAF1 (g) in cell subpopulations. ns: not significance * *P <* 0.05, ** *P <* 0.01, *** *P <* 0.001, **** *P <* 0.0001.

Supplementary Material 5. Communication of cell subpopulations (a-b) Communication number and strength of cell subpopulations in control (a) and AS samples (b).

Supplementary Material 6. Marker genes used for manual annotation of cell subpopulations.

## Data Availability

The datasets analysed during the current study are available from the GEO database (http://www.ncbi.nlm.nih.gov/geo/) [GSE43292, GSE100927, GSE11782, GSE159677].

## Abbreviations

AS: Atherosclerosis
CVD: Cardiovascular disease
MI: Myocardial infarction
ER: Endoplasmic reticulum
ERS: Endoplasmic reticulum stress
UPR: Unfolded protein response
ERAD: ER-associated degradation
ERSRG: Endoplasmic reticulum stress-related gene
DEG: Differentially expressed gene
WGCNA: Weighted gene co-expression network analysis
GO: Gene Ontology
KEGG: Kyoto Encyclopedia of Genes and Genomes
PPI: Protein-protein interaction
LASSO: Least absolute shrinkage and selection operator
SVM-RFE: Support vector machine-recursive feature elimination
ROC: Receiver operating characteristic
AUC: Area under the curve
GSEA: Gene set enrichment analysis
GSVA: Gene set variation analysis
TF: Transcription factor
lncRNA: Long noncoding RNA
miRNA: MicroRNA
scRNA-seq: Single-cell RNA sequencing
UMAP: Uniform manifold approximation and projection
VSMC: Vascular smooth muscle cell
EC: Endothelial cell
ox-LDL: Oxidized low-density lipoprotein
ASK1: Apoptosis signal-regulating kinase 1
MHC: Major histocompatibility complex
TRAIL: TNF-related apoptosis-inducing ligand
TBS: Tris-buffered saline
IKZF1: IKAROS family zinc finger 1
GEO: Gene Expression Omnibus
STRING: Search Tool for the Retrieval of Interacting Genes/Proteins
ChEA3: ChIP Enrichment Analysis 3
DSIGDB: Drug Signatures Database
FC: Fold change
PCA: Principal component analysis
