# Supplementary figures and images for: Identification and verification of diagnostic biomarkers related to endoplasmic reticulum stress for atherosclerosis
Source: BMC Cardiovasc Disord. 2026 Mar 20;26:368. doi: 10.1186/s12872-026-05745-5 (PMC13126753; doi:10.1186/s12872-026-05745-5)

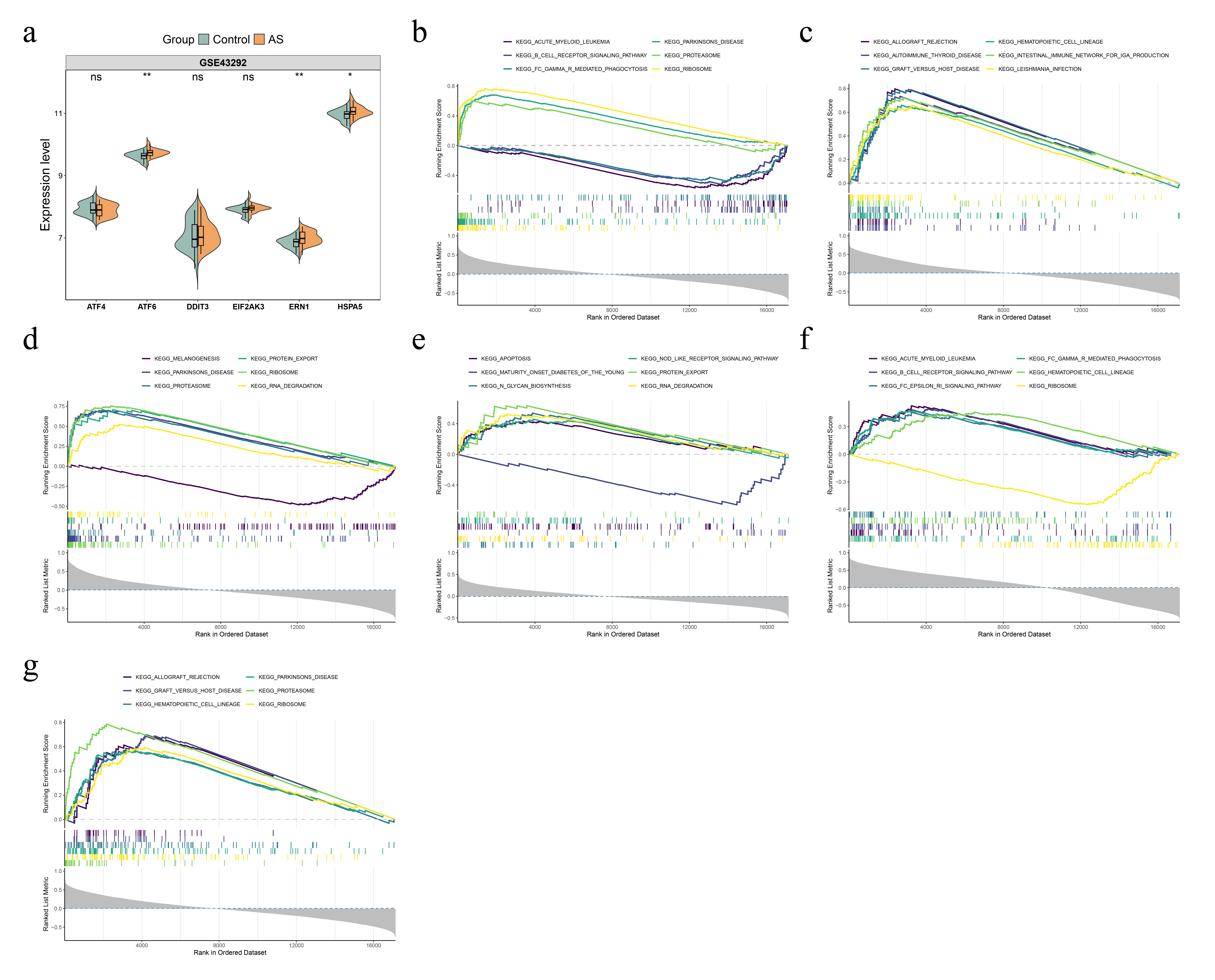

Supplement: Supplementary file 1 — Supplementary Material 1. Activation of canonical Endoplasmic Reticulum Stress (ERS)/Unfolded Protein Response (UPR) pathways in AS samples (a) Differential expression of key UPR markers between atherosclerotic and control samples (b-g) GSEA analysis of ATF4 (b), ATF6 (c), DDIT3 (d), EIF2AK3 (e), ERN1 (f), and HSPA5 (g). [file 12872_2026_5745_MOESM1_ESM.tif]

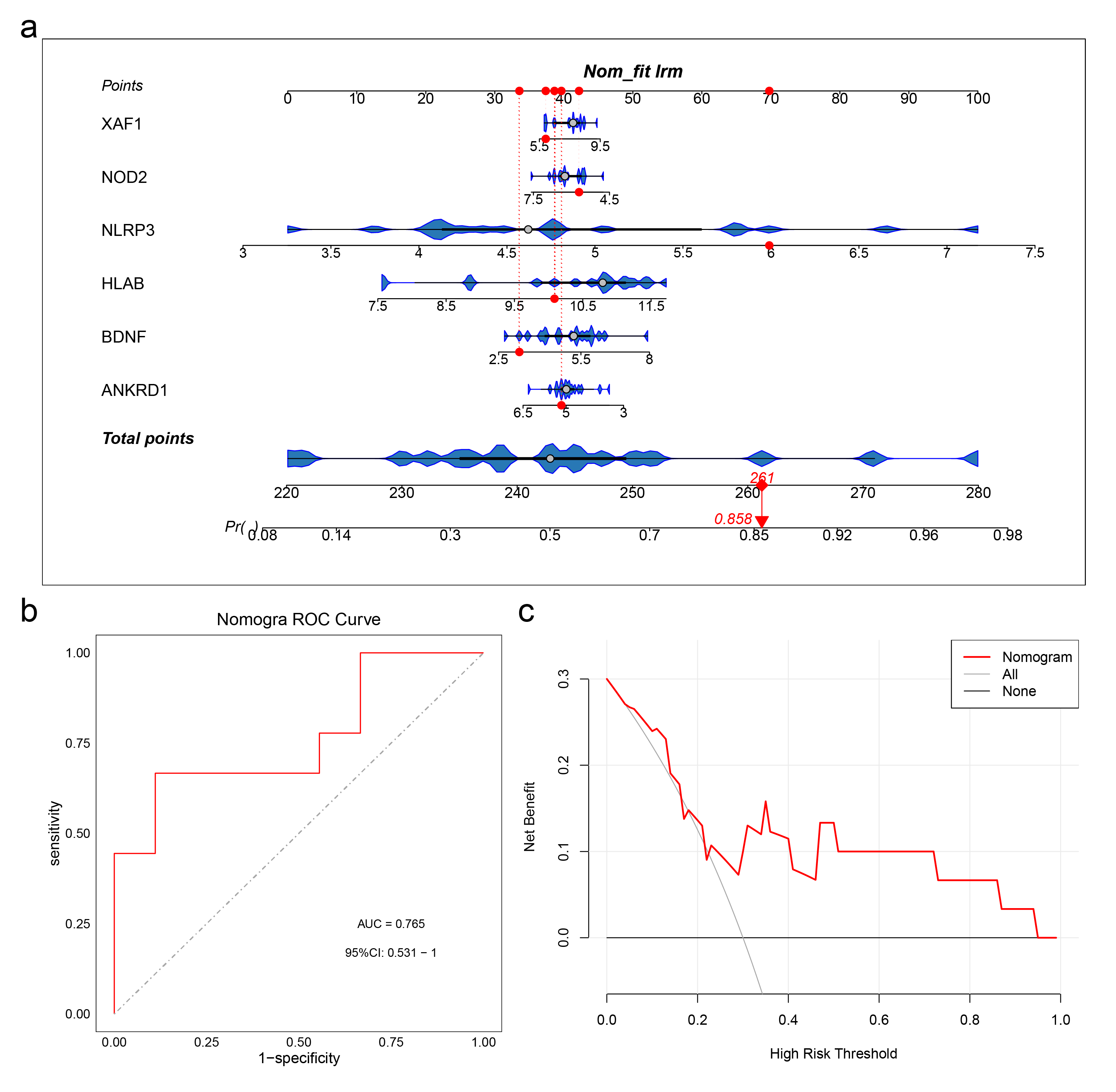

Supplement: Supplementary file 2 — Supplementary Material 2. Construction of nomogram for predicting AS occurrence (GSE111782) (a) Construction of nomogram model (b) ROC curve of nomogram. AUC, area under the curve; 95%CI, 95% confidence interval (c) Decision curve analysis (DCA) curve. [file 12872_2026_5745_MOESM2_ESM.tif]

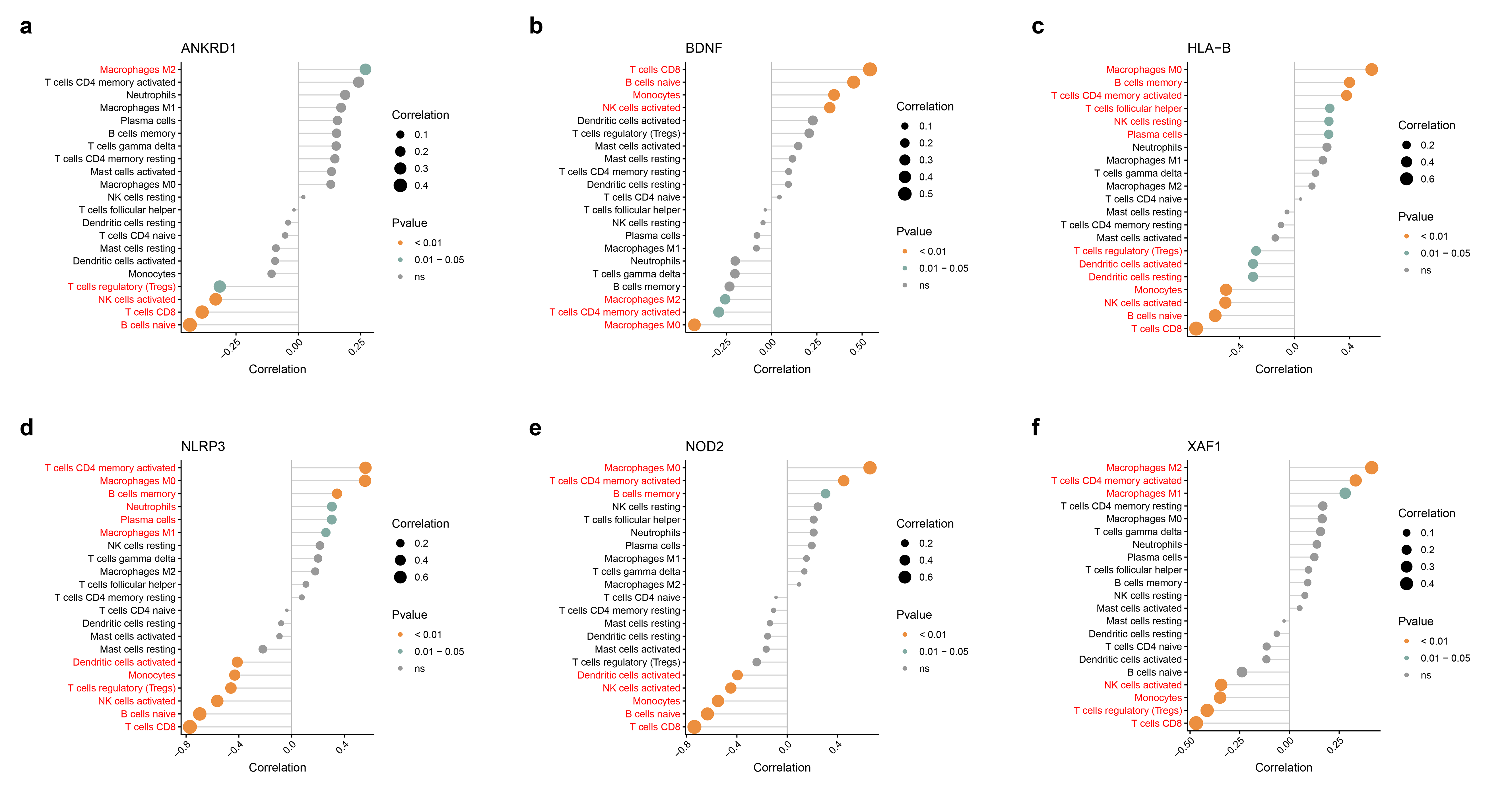

Supplement: Supplementary file 3 — Supplementary Material 3. The expression of biomarkers in immune cells (a-f) The correlation lollipop plot of immune cells with ANKRD1 (a), BDNF (b), HLA-B (c), NLRP3 (d), NOD2 (e), and XAF1 (f). [file 12872_2026_5745_MOESM3_ESM.tif]

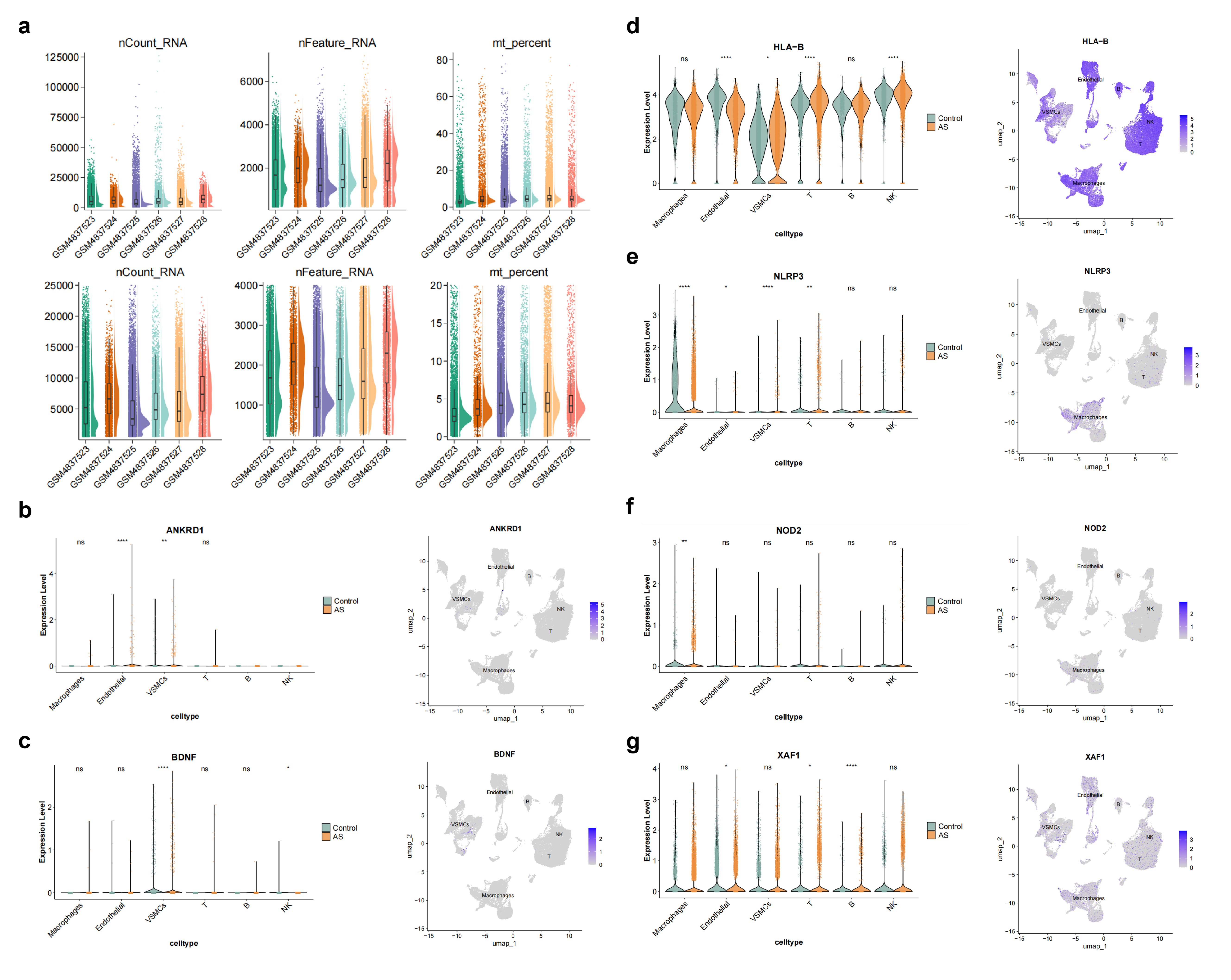

Supplement: Supplementary file 4 — Supplementary Material 4. Single-cell RNA sequencing (scRNA-seq) analysis in cell subpopulations (a) Quality control of data in GSE159677 dataset. (b-g) The expression of ANKRD1 (b), BDNF (c), HLA-B (d), NLRP3 (e), NOD2 (f), and XAF1 (g) in cell subpopulations. ns: not significance * P < 0.05, ** P < 0.01, *** P < 0.001, **** P < 0.0001. [file 12872_2026_5745_MOESM4_ESM.tif]

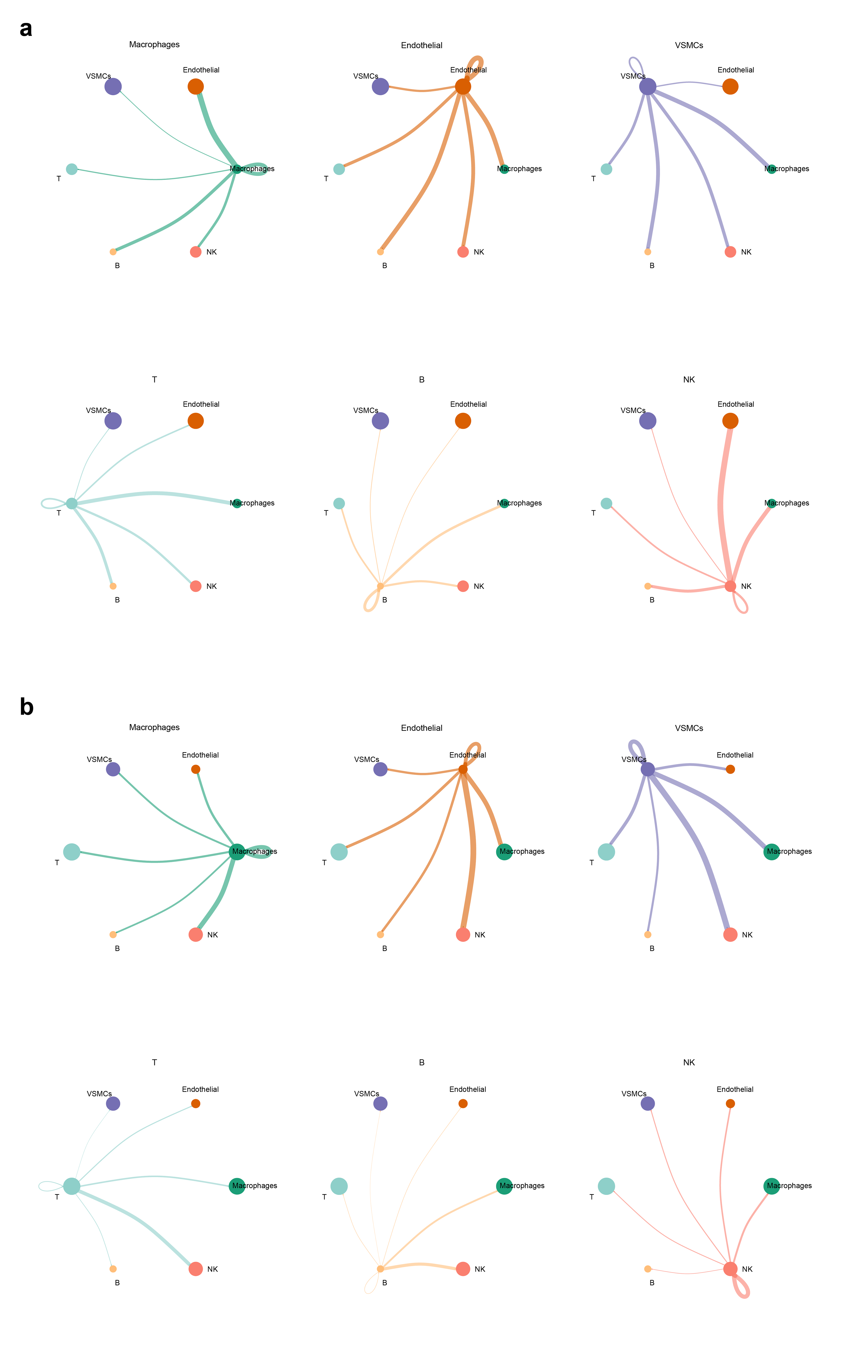

Supplement: Supplementary file 5 — Supplementary Material 5. Communication of cell subpopulations (a-b) Communication number and strength of cell subpopulations in control (a) and AS samples (b). [file 12872_2026_5745_MOESM5_ESM.tif]
